# Sarcosine sensitizes lung adenocarcinoma to chemotherapy by dual activation of ferroptosis via PDK4/PDHA1 signaling and NMDAR-mediated iron export

**DOI:** 10.1186/s40164-025-00657-0

**Published:** 2025-04-24

**Authors:** Guangyao Shan, Yunyi Bian, Shencheng Ren, Zhengyang Hu, Binyang Pan, Dejun Zeng, Zhaolin Zheng, Hong Fan, Guoshu Bi, Guangyu Yao, Cheng Zhan

**Affiliations:** 1 Department of Thoracic Surgery, Zhongshan Hospital, Fudan University, No. 180 Fenglin Road, Xuhui District, Shanghai, China; 2 Department of Thoracic Surgery, Zhongshan Hospital, Fudan University (Xiamen Branch), No. 668 Jinhu Road, Huli District, Xiamen, China

**Keywords:** Sarcosine, Ferroptosis, Lung adenocarcinoma, Chemotherapy, Organoids

## Abstract

**Background:**

Ferroptosis, a regulated cell death driven by iron-dependent lipid peroxidation, is associated with chemoresistance in lung adenocarcinoma (LUAD). This study aims to investigate the role of sarcosine in ferroptosis and its underlying mechanisms.

**Methods:**

An RSL3-induced ferroptosis model was used to screen a library of 889 human endogenous metabolites and metabolomic profiling was harnessed to identify metabolites associated with ferroptosis. Cell viability, lipid-reactive oxygen species (ROS), ferrous iron, malondialdehyde (MDA), and mitochondrial integrity were assessed to evaluate sarcosine’s effects on ferroptosis. Metabolic fate was studied using ^15^N-labeled sarcosine. Next, we used untargeted metabolomic profiling and next-generation sequencing to dissect metabolic and transcriptomic changes upon sarcosine supplementation. The effects of sarcosine on ferroptosis and chemotherapy were further validated in patient-derived organoids (PDOs), xenograft models, and LUAD tissues.

**Results:**

Sarcosine emerged as a potent ferroptosis inducer in the metabolic library screening, which was further confirmed via cell viability, lipid-ROS, ferrous iron, and MDA measurements. Metabolic flux analysis showed limited conversion of sarcosine to other metabolites in LUAD cells, while untargeted metabolomic profiling and seahorse assays indicated a metabolic shift from glycolysis to oxidative phosphorylation. Sarcosine enhanced pyruvate dehydrogenase activity to generate more ROS by interacting with PDK4, reducing PDHA1 phosphorylation. As a co-activator of N-methyl-D-aspartate receptor (NMDAR), sarcosine also exerted its pro-ferroptosis effect via regulating ferrous export through the NMDAR/MXD3/SLC40A1 axis. Given the significance of ferroptosis in chemotherapy, we validated that sarcosine enhanced the sensitization of cisplatin by promoting ferroptosis in LUAD cells, PDOs, and xenograft models.

**Conclusion:**

Sarcosine promotes ferroptosis and enhances chemosensitivity, suggesting its potential as a therapeutic agent in treating LUAD.

**Supplementary Information:**

The online version contains supplementary material available at 10.1186/s40164-025-00657-0.

## Background

Lung cancer, a highly prevalent malignancy, remains the leading cause of cancer-related mortality worldwide [1]. Notably, non-small cell lung cancer comprises approximately 85% of all lung cancer cases, with lung adenocarcinoma (LUAD) emerging as the most prevalent subtype [2]. Despite advancements in cancer therapy, the prognosis for LUAD patients remains dismal, with a five-year survival rate less than 26% [3].

Ferroptosis is a form of regulated cell death characterized by iron-dependent lipid peroxidation and subsequent membrane rupture [4]. In the realm of cancer therapeutics, the utilization of ferroptosis-inducing mechanisms has exhibited profound effectiveness in addressing resistance to conventional therapies, including targeted therapy, chemotherapy, and radiotherapy [5–7]. The biochemical hallmarks of ferroptosis include the generation of reactive oxygen species (ROS), the accumulation of lipid peroxides, an increase of intracellular ferrous iron, and the suppression of antioxidant systems [8]. Mitochondrial metabolic processes play a pivotal role in ferroptosis for electron leakage from complexes of the electron transport chain leads to the generation of ROS, primarily superoxides, which are then converted into hydrogen peroxide (H_2_O_2_) by superoxide dismutase. The subsequent reaction of H_2_O_2_ with labile iron via the Fenton reaction generates hydroxyl radicals, catalyzing lipid peroxidation and thus promoting ferroptosis. Furthermore, mitochondrial biosynthetic reactions, such as the tricarboxylic acid cycle (TCA cycle), have been identified as additional factors fostering ferroptosis via ROS production [9]. Despite the ambiguity surrounding the precise mechanisms of iron in ferroptosis, the pivotal role of iron metabolism in this process is unequivocal. Various regulators of iron metabolism, spanning from iron uptake, storage, and utilization, to efflux, are associated with the sensitivity to ferroptosis [10]. Notably, SLC40A1 is the only recognized transmembrane exporter of non-heme iron, the knockdown of which has been observed to promote ferroptosis [11].

Cancer cells exhibit metabolic adaptations that foster their accelerated growth and proliferation. However, these characteristics, including a high ROS burden and enhanced polyunsaturated fatty acid synthesis, inherently render some cancer cells vulnerable to ferroptosis [12]. Consequently, there is a pressing need to delve deeper into the interplay between tumoral metabolic traits and ferroptosis modulation, as well as to identify potential therapeutic targets for clinical application.

Sarcosine (N-methylglycine) is a metabolic intermediate in the glycine and choline pathways, playing a crucial role in one-carbon metabolism [13]. It functions dually as a competitive inhibitor of the type I glycine transporter and a co-agonist at the N-methyl-D-aspartate (NMDA) receptor. Preclinical and clinical studies have demonstrated sarcosine’s therapeutic potential, particularly as an adjunctive treatment for schizophrenia [14, 15]. In the context of cancer, sarcosine has gained attention as a potential biomarker, especially in prostate cancer, where elevated urinary levels are associated with increased tumor aggressiveness and metastatic potential [16]. Despite these findings, the roles of sarcosine in LUAD and its relationship with ferroptosis remain poorly understood.

In this research, we discovered that sarcosine markedly enhances LUAD cells’ susceptibility to ferroptosis through a comprehensive high-throughput screening of the metabolomics and endogenous metabolites library. This effect is mediated through a dual mechanism involving ROS production and iron export, highlighting sarcosine’s unique role in modulating ferroptosis. Furthermore, our findings revealed that sarcosine sensitizes LUAD to chemotherapy by inducing ferroptosis, thereby establishing it as a targetable vulnerability in cancer treatment.

## Methods and materials

### Cell lines and compounds

A549, PC9, MIAPACA2, HT-1080, and HEK-293T cell lines were obtained from the Chinese Academy of Sciences cell bank. Authentication of these cell lines was conducted using Short Tandem Repeat profiling to ensure their genetic identity and purity. Low passage cells (less than 30 passages) were used for experiments. The DMEM high glucose medium (KeyGEN BioTECH, Nanjing, China), supplemented with 10% fetal bovine serum (Lonsera, Shuangru Biotechnology Co., Ltd, Suzhou, China) or dialyzed fetal bovine serum (VivaCell, Shanghai, China), 0.1 mg/ml streptomycin, and 100 U/ml penicillin (Beyotime, Shanghai, China) was used for cell culture. Cells were cultured in a 37 °C incubator with a humidified 5% CO_2_ atmosphere.

The following compounds were procured from TargetMol (Boston, USA): sarcosine (T6975), imidazole ketone erastin (IKE, T5523), RAS-selective lethal 3 (RSL3, T3646), ferrostatin-1 (ferr-1, T6500), deferoxamine (DFO, T124358), Z-VAD (OMe)-FMK (T6013), necrosulfonamide (Necro, T7129), Dizocilpine (MK-801, T6259), Cisplatin (CDDP, dissolved in PBS, T1564), Staurosporine (T6680), and Rapamycin (T1537). Unless specified, all reagents were dissolved in DMSO to ensure proper solubility and stability.

### Untargeted metabolomic profiling

Cells were washed with ice-cold PBS three times. Subsequently, 1 mL extraction solution (2/2/1, v/v/v, methanol/acetonitrile/water) was added to the samples in 9-cm dishes and collected using cell scrapers and vortexed for 30 s. The sample was then frozen in liquid nitrogen for 1 min, thawed, and vortexed again for 30 s. This step was repeated three times. The sample was then sonicated in an ice-water bath for 10 min, followed by standing at -40 °C for 1 h. Subsequently, the sample was centrifuged at 1,3800 g for 15 min at 4 °C. 100 µL of the supernatant was transferred to a new EP tube and dried at 4 °C. The sample was then resuspended in 100 µL of methanol: acetonitrile: water (2:2:1, v/v/v) and sonicated in an ice-water bath for 10 min. Finally, the sample was centrifuged at 1,3800 g for 15 min at 4 °C. The supernatant was collected for test using the Vanquish UHPLC system (Thermo Fisher Scientific) and Orbitrap Exploris 120 Mass Spectrometer (Thermo Fisher Scientific). After converting the raw data into mzXML format using the ProteoWizard software, the metabolites were identified using the BiotreeDB (V3.0) database. All detections and analyses were performed by Biotree Biomedical Technology Co., Ltd (Shanghai, China).

### Metabolite library screening

The human endogenous metabolite library containing 889 compounds (HY-L030) was provided by MCE (Princeton, USA). LUAD cells were seeded in 96-well plates at a density of 2,500 cells per well. On the next day, cells were pre-incubated with DMSO or indicated metabolites for 12 h, followed by treatment with 2 µM RSL3 for 48 h. The cell viability was measured using CellTiter-Lumi™ Luminescent Cell Viability Assay Kit (Beyotime) on a microplate reader (SpectraMas iD3, Molecular Devices, San Jose, USA).

### Cytotoxicity assays

Cells were seeded at an appropriate density in 96-well plates, with each well containing a total volume of 100 µL of culture media. The plates were then incubated overnight to ensure cell adherence. Following exposure to the indicated treatments, cell viability was assessed using the Cell Counting Kit-8 (CCK8, TargetMol) following the manufacturer’s recommended protocols. The absorbance of each well was subsequently measured at 450 nm using a microplate reader.

### Detection of lipid peroxidation

The measurement of lipid peroxidation was performed as previously reported [17]. In brief, cells were seeded onto 12-well plates and allowed to adhere overnight. Subsequently, the cells were treated as indicated and harvested via trypsinization. After being washed with PBS solution, the cells were resuspended in fresh media containing 4 µM of the BODIPY 581/591 C11 fluorescent probe (Thermo Fisher Scientific, Waltham, USA) and then incubated at 37 °C for 30 min. After removing the unbound dye with PBS solution, the lipid peroxidation levels were then quantified using an Accuri C6 flow cytometer (BD Biosciences, San Diego, USA) with a 488-nm laser. The results were analyzed using FlowJo software (TreeStar, Woodburn, USA).

### Assessment of intracellular ferrous iron and malondialdehyde (MDA)

To evaluate intracellular ferrous iron levels, we employed the Iron Assay Kit (Dojindo Molecular Technologies, Inc., Kumamoto, Japan) and FerroOrange probes (Dojindo Molecular Technologies, Inc.), following the manufacturer’s protocols. For the Iron Assay Kit, optical density measurements were obtained at a wavelength of 593 nm using a microplate reader. The fluorescence intensity of FerroOrange probes was quantified utilizing an Accuri C6 flow cytometer through the PE channel.

The MDA levels of cell lysates were analyzed using the MDA Assay Kit (Beyotime) based on the reaction between MDA and thiobarbituric acid, which generates a red product. The absorbance was colorimetrically detected at 532 nm.

### Transmission electron microscopy

The samples were prepared as previously reported [18]. Cells were seeded onto 6-cm dishes and subjected to various treatment conditions. Following treatment, the cells were fixed in a 2.5% glutaraldehyde solution and rinsed three times with 0.1 mM phosphate buffer (pH 7.4). Subsequently, the cells were postfixed in phosphate buffer containing 1% osmic acid and washed an additional three times. After postfixation, the samples underwent dehydration and embedding procedures and then were cured in an oven at 60 °C for 48 h. Ultrathin sections were prepared from the embedded samples and subsequently stained with lead citrate and uranyl acetate. After drying overnight, the ultrathin sections were observed under a transmission electron microscope (Hitachi, Tokyo, Japan).

### Metabolic flux assay and data analysis

[^15^N-Sarcosine]-containing DMEM was prepared by supplementing a final concentration of 500 µM ^15^N-Sarcosine (HY-101037S, MCE). A549 cells were seeded into 10 cm plates. After the cells reached approximately 50% confluence, the normal DMEM was replaced with [^15^N-Sarcosine]-containing DMEM for 48 h of incubation. Then, the cells were washed with cold PBS three times and were collected by cell scrapers using 1 ml cold extraction solution (methanol: acetonitrile: H_2_O = 2:2:1, v/v/v). After vortexing for 30 s, the samples were frozen with liquid nitrogen and thawed three times. Subsequently, the samples were sonicated for 10 min in an ice-water bath. Then, the samples were incubated at -40 °C for 1 h and centrifuged at 12,000 rpm for 15 min at 4 °C. 800 µL supernatant of each sample was transferred to a new EP tube and dried at 4 °C, followed by reconstitution with 100 µL mix of acetonitrile: H_2_O (1:1, v/v). The samples were further sonicated in an ice-water bath for 10 min and then centrifuged at 12,000 rpm at 4 °C for 15 min. The supernatant was transferred to a glass vial for metabolite profiling using the Vanquish UHPLC system (Thermo Fisher Scientific) and Orbitrap Exploris 120 Mass Spectrometer (Thermo Fisher Scientific). The raw data were converted to the mzXML format using ProteoWizard software and were annotated based on the BiotreeDB(V3.0)database [19]. This experiment was performed by Biotree Biomedical Technology Co., Ltd.

### ROS detection

The generation of ROS and mitochondrial ROS were assessed utilizing a ROS Assay Kit (Beyotime) and MitoSOX Red (Beyotime), respectively. After exposure to various treatments as indicated, the cells were subjected to staining with 10 µM of DCFH-DA and 5 µM MitoSOX Red (derivatives of Hydroethidine) at 37 °C for 20 min and then captured using a fluorescence microscope (Olympus IX71, Olympus, Tokyo, Japan).

### Measurement of pyruvate, citrate, lactate, sarcosine, ATP production and PDH activity

The pyruvate, citrate, lactate, ATP production, sarcosine, and PDH activity were quantitatively assessed using the Pyruvate Content Assay Kit (BC2205, Solarbio, Beijing, China), Citric Acid Content Assay Kit (BC2155, Solarbio), Lactic Acid Content Assay Kit (BC2235, Solarbio), ATP Content Assay Kit (BC0300, Solarbio), Sarcosine Assay Kit (S486337, Aladdin, Shanghai, China) and PDH Activity Assay Kit (BC0385, Solarbio) following the instructions of each manufacturer, respectively. The optical density of the extracted samples was determined spectrophotometrically using a microplate reader.

### Western blot

Cell lysis was prepared using cold RIPA buffer (Beyotime) supplemented with a protease inhibitor and two phosphatase inhibitor cocktails (TargetMol). Protein concentrations were quantified using the BCA Protein Quantification Kit (YEASEN). An amount of 25 µg protein was loaded onto SDS-PAGE gels. Following electrophoresis, the proteins were transferred onto PVDF membranes (Millipore, Burlington, USA). After being blocked with 5% non-fat milk at room temperature for 1 h, the membranes were then incubated with indicating primary antibodies overnight at 4 °C. After thorough washing with Tris-buffered saline-Tween solution to remove unbound antibodies, the membranes were incubated with HRP-conjugated secondary antibodies (1:2,500, Beyotime) for 1 h at room temperature. Subsequently, BeyoECL Moon Chemiluminescence Reagent (Beyotime) was used to detect the protein bands. Details of the primary antibodies were provided in Supplementary Table 1.

### Molecular Docking

The protein crystal structures of PDK1 (2Q8F), PDK2 (1JM6), PDK3 (2Q8I), and PDK4 (3D2R) were retrieved from RCSB PDB (https://www.rcsb.org/) and the molecular structures of alanine (CID:5950) and sarcosine (CID: 1088) were downloaded from PubChem (https://pubchem.ncbi.nlm.nih.gov/). The potential interactions between isolated compounds and their respective enzyme binding sites were systematically investigated using the Molecular Operating Environment (MOE) software (Chemical Computing Group Inc., Montreal, Canada). Prior to docking, the ligands underwent an “energy minimization” step to eliminate any potential biases in bond lengths and bond angles to ensure the accuracy of the subsequent docking simulations.

### Cell energy metabolic analysis

Glycolysis stress and mitochondrial stress tests were conducted to quantify the extracellular acidification rate (ECAR) and oxygen consumption rate (OCR) in LUAD cells using a Seahorse XFe24 flux analyzer (Agilent Seahorse Bioscience, Santa Clara, USA). A549 or PC9 cells were cultivated in a Seahorse XF96 plate at a density of 30,000 cells per well, and allowed to grow overnight. Cells were treated with sarcosine (0.5 mM) for 24 h. Subsequently, the growth medium was replaced with XF assay medium, and the cells were incubated in a CO_2_-free environment for 1 h before measurement. Each experimental measurement involved four sequential injections. For the glycolysis stress test, the final concentrations of reagents were as follows: 10 mM glucose, 2 µM oligomycin, and 50 mM 2-deoxy-D-glucose (2-DG) for each group. In the mitochondrial stress test, the final concentrations of reagents were 2 µmol/L oligomycin, 1 µM FCCP (trifluoromethoxy carbonylcyanide phenylhydrazone), and 0.5 µM antimycin A/rotenone for each group.

### RNA sequencing

To investigate the transcriptional response of A549 cells to sarcosine, total RNA was extracted using the TRIzol reagent (TIANGEN). Sequencing was conducted by OE Technology (Shanghai, China), and the raw data were normalized to Fragments Per Kilobase of exon model per Million mapped fragments (FPKM) format for subsequent analysis.

### Lentivirus transfection and CRISPR/Cas9-mediated gene knockout

Lentivirus vectors of MXD3-SH1, MXD3-SH2, 1xFlag-MXD3, PDK4, PDK4-ΔAdom3 and negative control were created by Genechem (Shanghai, China). Cells were transfected with lentivirus using HiTrans A (Genechem) following the manufacturer’s protocol and screened by puromycin (Beyotime) for 48 h. CRISPR/Cas9 technology was utilized to achieve targeted gene knockout in cells. Specifically, sgRNAs targeting PDK4 or SLC40A1 were designed and cloned into GV392 plasmids, which also contained the puromycin-resistant gene and the hSpCas9 gene. Subsequently, these modified vectors were packaged into lentiviruses in targeted cells. The entire process of vector design, construction, and lentivirus packaging was carried out by Genechem. The information of shRNAs and sgRNAs was provided in Supplementary Table 1.

### RNA isolation and reverse transcription-quantitative polymerase chain reaction (RT-qPCR)

Total RNA extraction was conducted utilizing the TRIzol reagent (TIANGEN, Beijing, China). Subsequently, the isolated RNA was reverse-transcribed into cDNA using the Hifair II First-Strand cDNA Synthesis Kit (YEASEN, Shanghai, China). RT-qPCR was performed using the Hieff qPCR SYBR Green Master Mix (YEASEN) on an ABI QuantStudio 5 real-time PCR system (Thermo Fisher Scientific). The experimental samples were assayed in triplicate, and the relative mRNA expression levels were determined using the 2^−ΔΔCT^ method, with β-actin serving as an endogenous reference gene for normalization. All primers utilized in this investigation were provided in Supplementary Table 1.

### Patient selection

We collected the resected tumor specimens and matched clinical data from 120 LUAD patients who underwent surgical resection at Zhongshan Hospital, Fudan University (February 2016–August 2023), including serum samples from 100 patients. A subset of 41 patients received cisplatin-based chemotherapy. Surgical resections were performed by board-certified thoracic surgeons, with histopathological diagnoses independently verified by two certified pathologists. Tumor staging followed the 8th edition AJCC TNM classification system. All procedures adhered to the Declaration of Helsinki and were approved by the Zhongshan Hospital Research Ethics Committee (No. B2022-180R).

### Immunohistochemistry (IHC)

The formalin-fixed and paraffin-embedded tissue samples from LUAD patients were collected. The slides underwent overnight baking at 65 °C, followed by three sequential dewaxing steps in xylene, each lasting 15 min. Rehydration was achieved through immersion in a graded alcohol series. Antigen retrieval was performed using citrate buffer (Sangon Biotech, Shanghai, China), and the slides were subsequently blocked with peroxidase blocking buffer at 37 °C for 30 min to minimize non-specific binding. After a 10-minute incubation in blocking buffer, the slides were incubated with primary antibodies overnight at 4 °C, followed by incubation with a secondary antibody for 30 min at room temperature. The staining was achieved using the GTVisionTM III Detection System/Mo&Rb (GeneTech, Shanghai, China) according to the manufacturer’s instructions. The primary antibodies utilized for IHC staining were listed in Supplementary Table 1.

For quantitative analysis of IHC staining, four random fields of view were selected from each slide. The staining intensity was graded on a scale of 0 (no staining) to 3 (strong staining), while the staining area was scored based on the percentage of positive cells (0 for ≤ 5%, 1 for 5–25%, 2 for 26–50%, 3 for 51–75%, and 4 for > 75%). A histological score was derived by multiplying the staining intensity and area scores. This score was then categorized as negative (−, score 0), weakly positive (+, score 1–4), moderately positive (++, score 5–8), or strongly positive (+++, score 9–12). For MXD3, low expression was defined as a score of − or +, while + + and +++ were classified as high expression.

### Chromatin immunoprecipitation (ChIP) assay

We conducted ChIP assays using the SimpleChIP Plus Enzymatic Chromatin IP Kit (Cell Signaling Technology, Danvers, USA) as previously reported [20]. Cells were subjected to crosslinking, membrane lysis, and enzymatic digestion with micrococcal nuclease, generating DNA/protein fragments ranging from 150 to 600 bp. These DNA/protein fragments were incubated overnight at 4 °C with anti-flag (1:50, 14793T, Cell Signaling Technology) or IgG. Protein G magnetic beads were then added to capture the immunoprecipitated complexes for a further overnight incubation at 4 °C. After elution of the chromatin, cross-links were reversed, and the resulting DNA fragments were purified using spin columns. Finally, the quantity of immunoprecipitated DNA fragments was determined using qRT-PCR. The primers were listed in Supplementary Table 1.

### Dual-luciferase reporter assay

The promoter sequence of SLC40A1 and the corresponding mutated sequence on the predicted target sites were cloned into the firefly luciferase reporter plasmid vectors by Genechem. These plasmids were then transfected into Ctrl or MXD3-overexpressed 293T cells using the lipo8000 Transfection Reagent (Beyotime). In addition, renilla luciferase reporter plasmids were co-transfected. Dual-luciferase reporter assays were performed 48 h post-transfection using the Luciferase Reporter Gene Assay Kit (Beyotime).

### Establishment of patient-derived organoids (PDOs)

Fresh LUAD tumor tissues were processed for the establishment of PDOs. The tissues were dissected into fragments, rinsed with ice-cold PBS supplemented with 0.3 mg/ml streptomycin and 300 U/ml penicillin three times, and enzymatically digested using organoid digestive solution (D1Med, Shanghai, China). Following filtration and centrifugation, the cell precipitate was resuspended in a 25-fold volume of matrigel (YEASEN) and seeded into a 24-well plate. After gelation at 37 °C for 10 min, 500 µL of human LUAD-PDO culture medium (D1Med) was added to each well to facilitate the growth of PDOs. For histological analysis, PDOs were harvested and fixed in 4% paraformaldehyde. Subsequently, they were embedded in agarose and paraffin, sectioned, and subjected to standard hematoxylin and eosin staining procedures, including deparaffinization, dehydration, and staining. To assess drug sensitivity, PDOs were plated in 96-well plates and treated with indicating agents. Cell viability was measured using the CellTiter-Lumi Luminescent 3D Cell Viability Assay Kit (Beyotime).

### Cell-derived xenograft model

This research was conducted following the ethical guidelines of the Research Ethics Committee of Zhongshan Hospital, Fudan University. BALB/c nude mice were procured from GemPharmatech (Nanjing, China) and housed in pathogen-free laminar flow conditions. A total of 2 × 10^6^ Ctrl or PDK4-OE A549 cells were resuspended in 100 µL of PBS solution and administered via subcutaneous injection into the right flank of each nude mouse. Sarcosine (2 mM) was orally administered through drinking water, while MK-801 was intraperitoneally injected at a dose of 0.05 mg/kg. Once tumors attained a volume of approximately 100 mm^3^, the mice were randomly allocated into three distinct groups (*n* = 5 per group) for treatment with IKE (40 mg/kg), CDDP (3 mg/kg), or normal saline. These reagents were administered intraperitoneally every three days for a total of six treatments. It was noted that none of the treatment regimens had a significant impact on the body weight of the mice. Tumor sizes were monitored weekly using vernier calipers, and after 4 weeks, the mice were euthanized. Tumor volumes were calculated using the formula: (length × width^2^) / 2. To minimize potential biases, the investigators were blinded to the allocation during outcome assessment.

### Multiplex IHC

The processing of each section was conducted as aforementioned for IHC, including paraffin dissolution, dewaxing in xylene, rehydration, antigen retrieval, cooling, permeabilization, and blocking. Subsequently, the sections underwent overnight incubation with primary antibodies at 4 °C, followed by washing with PBS to remove unbound antibodies. Subsequently, the sections were incubated with corresponding secondary antibodies for 1 h and then treated with Tyramide signal amplification (TSA) reagents (Servicebio, Wuhan, China) for 10 min at room temperature. To remove primary and secondary antibodies that had already bound to the tissue, the sections were placed in a box containing citrate repair solution (pH = 6.0) and heated in a microwave oven to maintain boiling conditions for 10 min. This blocking step was repeated, followed by a second application of primary antibodies, and subsequent incubation with secondary antibodies and TSA reagents. For nuclear visualization, the slides were stained with DAPI (Servicebio). TSA agents were listed as follows: iF440-Tyramide (1:500), iF488-Tyramide (1:500), iF546-Tyramide (1:500), iF647-Tyramide (1:500). The primary antibodies employed for each staining procedure were listed in Supplementary Table 1.

### Single-cell sequencing and data processing

The Single-cell data of 29 samples, including 12 normal lung tissues and 17 LUAD tissues were collected as previously reported [21]. Subsequently, we conducted quality control, dimensionality reduction, and unsupervised clustering analysis to identify cell types and explore the gene expression patterns.

### Statistical analyses

Statistical analysis was conducted using unpaired two-tailed Student’s t-test or one-way analysis of variance (ANOVA) with FDR adjustment as appropriate. The Spearman correlation was conducted using the R cor.test function and visualized using the R ggplot2 package. The statistical analysis and visualization were conducted in R (Version 4.1.1, R Foundation for Statistical Computing, Vienna, Austria) and GraphPad Prism 9 (GraphPad Software, La Jolla, USA). In all statistical tests, P values less than 0.05 were considered as indicative of statistical significance.

## Results

### Metabolite library screening links sarcosine to ferroptosis

**Fig. 1:**
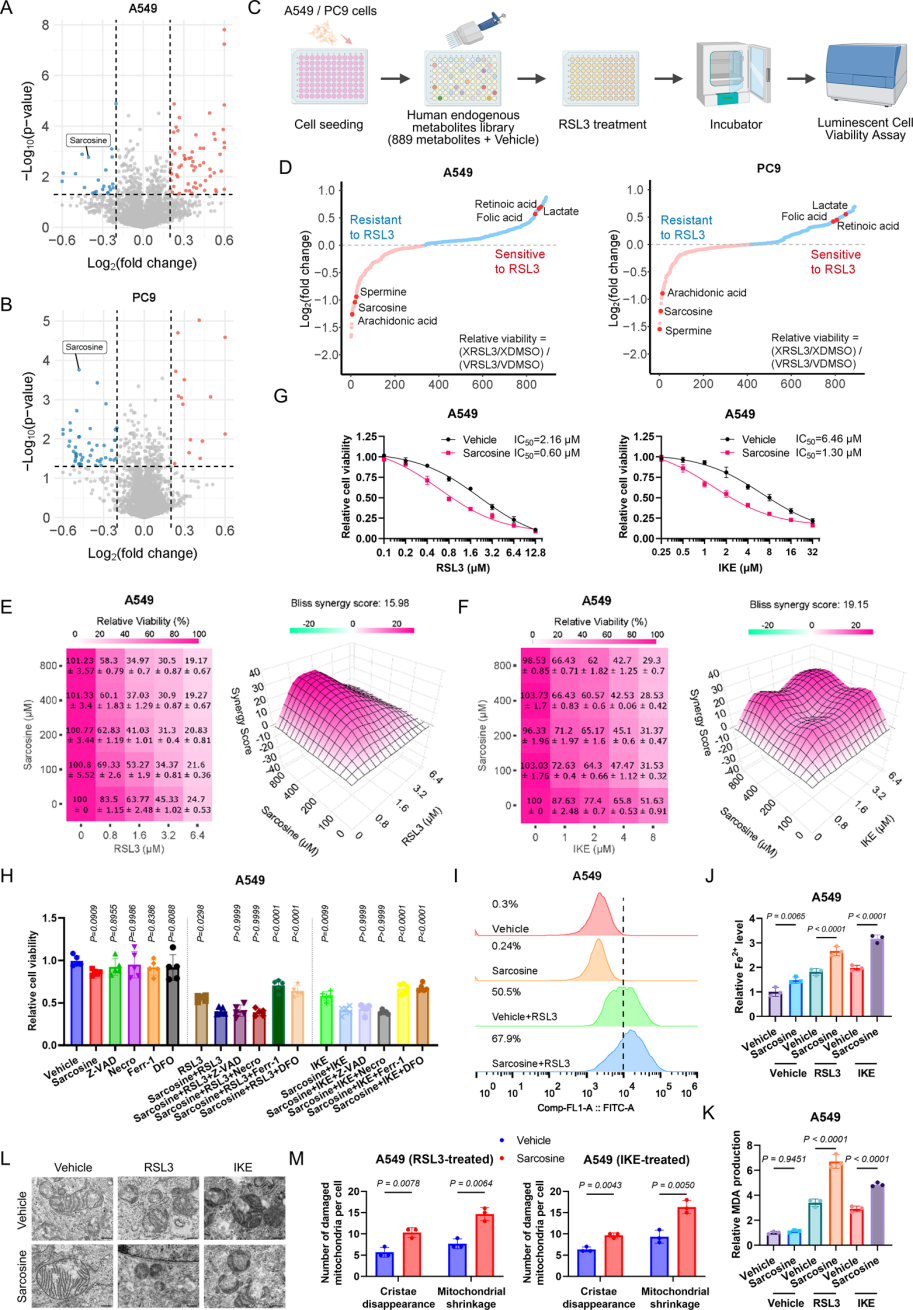
Metabolite library screening links sarcosine to ferroptosis. **(A-B)** Volcano plots of differentially expressed metabolites in A549 (**A**) and PC9 (**B**) cells treated with RSL3 (2 µM, 48 h). Thresholds: |log2FC| >0.2, -log10(P) > 1.301. **(C)** Workflow diagram for metabolite library screening. **(D)** The metabolite library screening results were demonstrated as the relative viability of metabolite-treated (X) cells versus DMSO-treated (vehicle, V) cells. LUAD cells pretreated with metabolites (24 h) followed by RSL3 (2 µM, 48 h; *n* = 5). **(E-F)** Bliss synergy scores for sarcosine combined with RSL3 (48 h) or IKE (72 h) in A549 cells. **(G)** Viability curves of sarcosine (0.5 mM)-treated A549 cells exposed to RSL3/IKE (*n* = 4). **(H)** Rescue effects of cell death inhibitors (10 µM each) on sarcosine-potentiated ferroptosis (*n* = 5). **(I-K)** Lipid-reactive oxygen species (lipid-ROS), ferrous iron, and malondialdehyde (MDA) levels in cells pretreated with sarcosine (0.5 mM, 24 h) followed by RSL3 (2 µM) or IKE (10 µM) for 8 h. **(L-M)** Transmission electron micrographs **(L)** and quantification **(M)** of mitochondrial ultrastructure. Scale bars, 4 μm. Data were presented by mean (SD) and analyzed by one-way analysis of variance (ANOVA) with FDR adjustment. P-value less than 0.05 was considered as significant

In our investigation of the metabolites underlying ferroptosis, we conducted untargeted metabolomic profilings of A549 and PC9 cell lines, revealing a distinct metabolic signature characterized by the downregulation of sarcosine and upregulation of six other metabolites (Fig. 1A & B). Subsequently, we embarked on a comprehensive screening of 889 human endogenous metabolites within RSL3-induced ferroptotic models of A549 and PC9 cells, which also pinpointed sarcosine as a potent enhancer of RSL3-mediated ferroptosis (Fig. 1C & D).

To further validate this finding, we conducted cytotoxicity assays in A549 and PC9 cells, which demonstrated a synergistic effect of sarcosine with ferroptosis inducers (FINs) (Fig. 1E – G, S1A - D). Besides, the sensitization of LUAD cells to FINs by sarcosine was completely abolished by ferroptosis inhibitors, ferrostatin-1 (Fer-1, a lipid peroxidation scavenger) and deferoxamine (DFO, an iron chelator), while remaining unaffected by apoptosis (Z-VAD-FMK) or necroptosis (necrosulfonamide) inhibitors, thereby confirming the specificity of sarcosine’s role in promoting ferroptosis (Fig. 1H, S1E). Since lipid peroxidation is a hallmark of ferroptosis, we employed BODIPY-C11 581/591 probes to show that exogenous sarcosine significantly potentiated RSL3-induced lipid peroxidation (Fig. 1I, S1F). Concordantly, sarcosine-treated cells exhibited elevated ferrous iron levels and MDA, a lipid peroxidation biomarker (Fig. 1J & K, S1G & H). Transmission electron microscopy further demonstrated exacerbated mitochondrial damage in sarcosine-exposed A549 cells, characterized by more pronounced cristae disappearance and mitochondrial shrinkage (Fig. 1L & M). Consistent with these observations, sarcosine promoted ferroptosis in MIAPACA2 and HT-1080 cells (Fig S1I), while exhibiting no significant impact on autophagy or apoptosis (Fig S1J & K). Collectively, these findings indicated that sarcosine enhances cellular susceptibility to ferroptosis.

### Sarcosine rewires cellular metabolism to potentiate ferroptosis

**Fig. 2:**
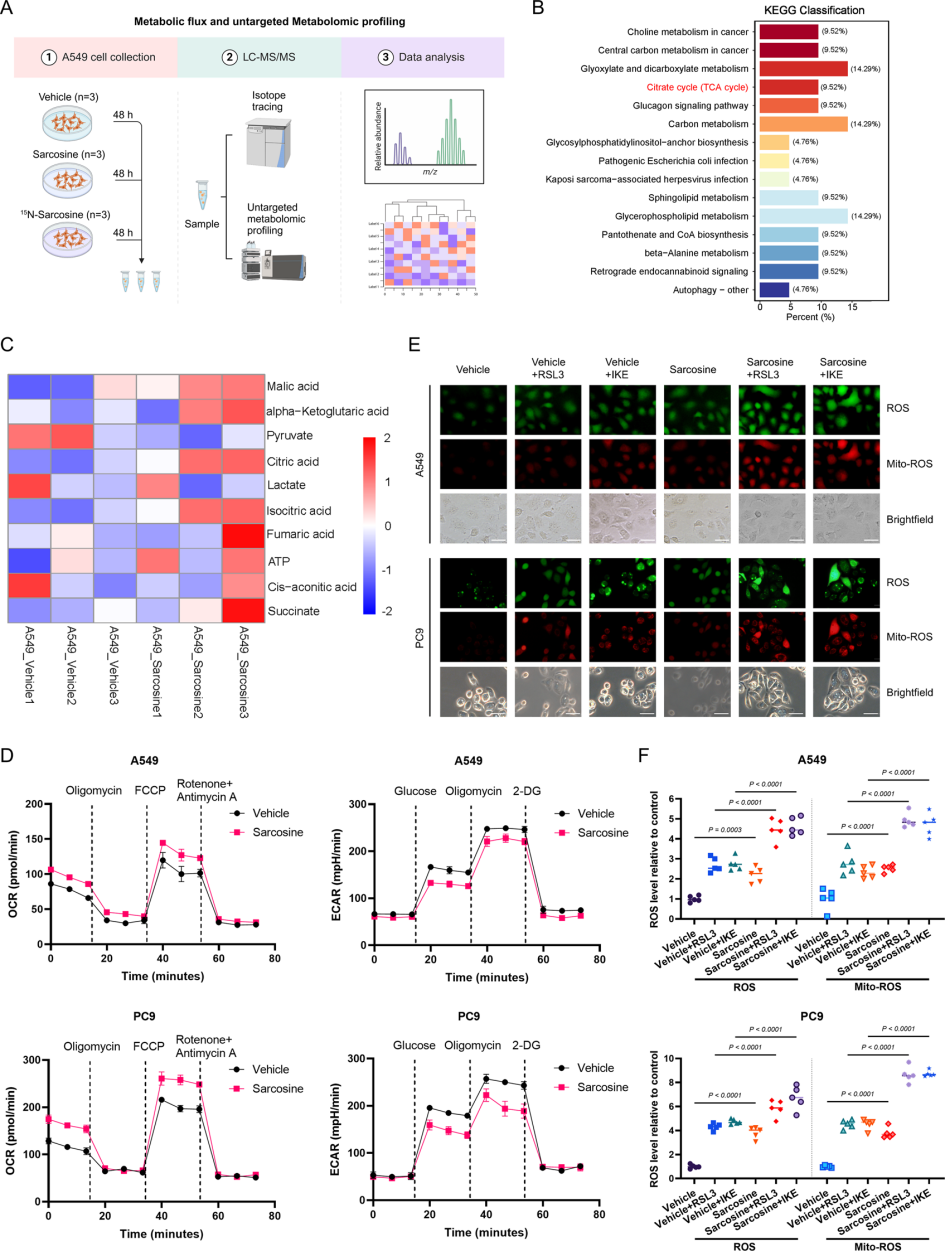
Sarcosine rewires cellular metabolism to potentiate ferroptosis. **(A)** Experimental design for ^15^N-sarcosine tracing and metabolomic profiling. **(B)** KEGG pathway enrichment of differential metabolites (TCA cycle highlighted). **(C)** Heatmap of TCA cycle-related metabolites in sarcosine-treated (0.5 mM, 48 h) A549 cells. **(D)** Oxygen consumption rate (OCR) and extracellular acidification rate (ECAR) measured by Seahorse mitochondrial/glycolysis stress tests (*n* = 3). **(E-F)** Fluorescence images **(E)** and quantification **(F)** of cellular reactive oxygen species (ROS) and mitochondrial ROS (MitoROS) in sarcosine-pretreated (0.5 mM, 24 h) cells exposed to RSL3 (2 µM) or IKE (10 µM) for 8 h. Scale bars, 50 μm. Data were presented by mean (SD) and analyzed by Student’s t-test or one-way ANOVA with FDR adjustment. P-value less than 0.05 was considered as significant

In subsequent investigation, we focused on the mechanism underlying sarcosine’s role in mediating ferroptosis. Sarcosine, as a derivative of glycine in animal cells, can be metabolized to glycine in the presence of SARDH or PIPOX (Fig S2A). To elucidate the metabolic trajectory of sarcosine, we labeled the sarcosine in DMEM medium with a ^15^N isotope (^15^N-sarcosine) to track its metabolic fate via liquid chromatography-mass spectrometry (Fig. 2A). However, the isotope results showed that sarcosine undergoes minimal conversion to other metabolites, including glycine, in A549 cells (Fig S2B & C, Supplementary material 1). Given that glycine is a crucial constituent of GSH, we also examined the GSH/GSSG ratio in LUAD cells, which revealed no significant difference upon sarcosine supplementation (Fig S2D), thereby reassuring us that sarcosine does not exert a substantial impact on glycine metabolism in LUAD cells. To further corroborate these findings, we interrogated the RNA-seq data from the Cancer Cell Line Encyclopedia (CCLE) database and observed that SARDH, PIPOX, and GNMT, which are responsible for the conversion between glycine and sarcosine, were scarcely expressed in commonly used LUAD cell lines (Fig S2E).

Based on these findings, we postulated that the intracellular sarcosine present in A549 and PC9 cells likely originates from the serum component of the culture media. To this end, we substituted the serum in the culture media with dialyzed serum (small molecules with molecular weights below 10,000 Daltons were removed) and found that the intracellular sarcosine was hardly detected (Fig S3A). Conversely, supplementation with sarcosine restored its intracellular concentrations, corroborating our speculation that A549 and PC9 cells are incapable of de novo synthesis or substantial metabolism of sarcosine.

Subsequently, we performed untargeted metabolomics profiling to assess how sarcosine impairs cellular resistance to ferroptosis. Our KEGG enrichment analysis revealed that the differentially expressed metabolites were predominantly enriched in the TCA cycle (Fig. 2B). Detailed examination of the TCA cycle metabolites showed a significant decrease in pyruvate levels and an increase in citrate levels upon sarcosine supplementation (Fig. 2C). Utilizing specialized assay kits, we confirmed that both citrate and ATP production were elevated, whereas pyruvate and lactate contents were reduced in LUAD cells (Fig S3B). Furthermore, seahorse assays indicated that sarcosine induced a metabolic shift from glycolysis toward oxidative phosphorylation (Fig. 2D). By employing ROS and mitochondrial ROS (Mito-ROS) probes, we observed that sarcosine treatment led to an increase in ROS generation, particularly within the mitochondria (Fig. 2E & F).

Together, these data revealed that sarcosine drives ferroptosis, at least partially, through metabolic rewiring from glycolysis to oxidative phosphorylation, which amplifies mitochondrial ROS and disrupts redox homeostasis, ultimately priming LUAD cells for ferroptosis.

### Sarcosine inhibits PDK4 to drive TCA cycle activation

**Fig. 3:**
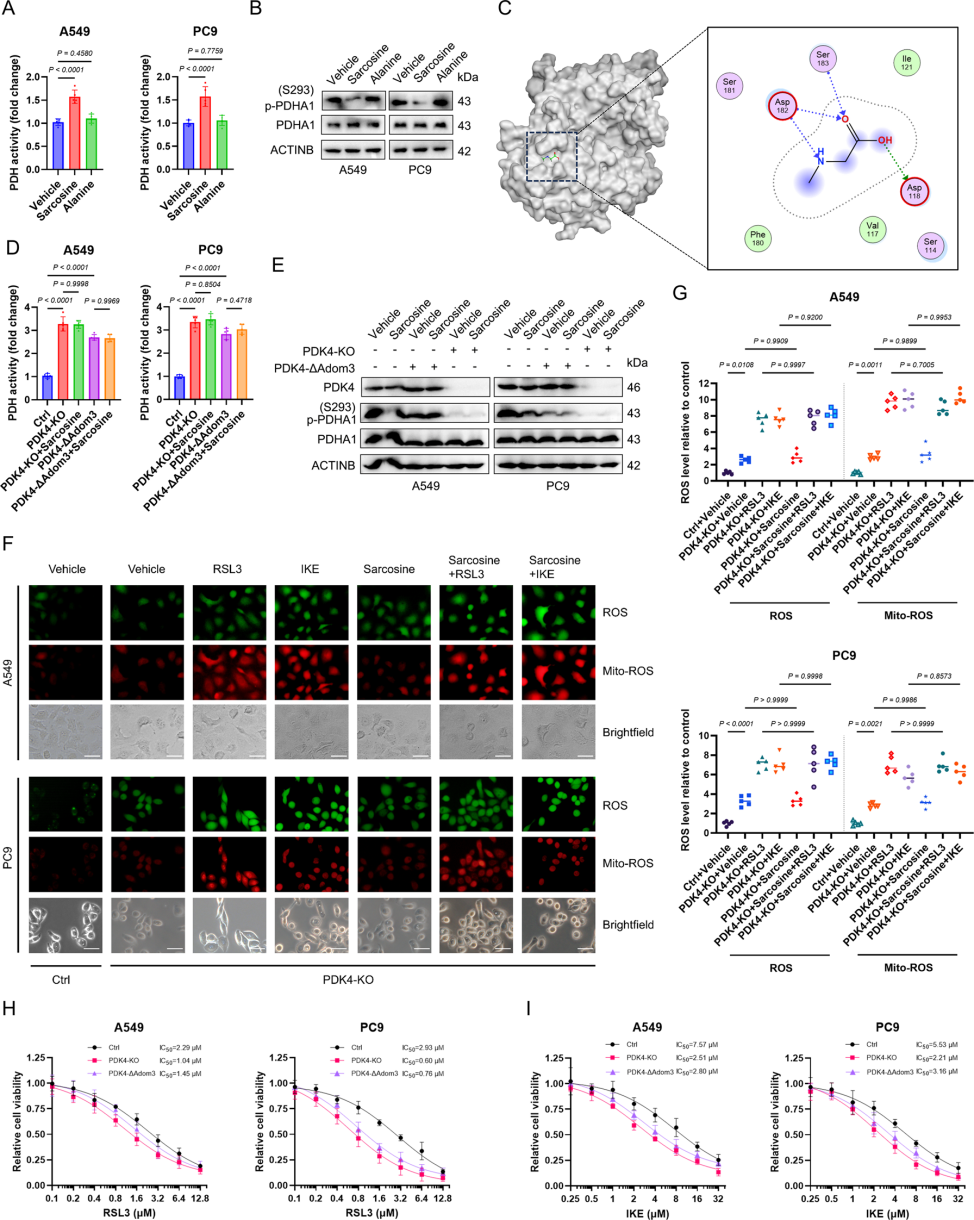
Sarcosine inhibits PDK4 to drive TCA cycle activation. **(A)** PDH activity in LUAD cells treated with sarcosine/alanine (0.5 mM, 48 h). **(B)** Immunoblot analysis of PDHA1 phosphorylation (S293) in sarcosine or alanine-treated cells (0.5 mM, 48 h). **(C)** Predicted sarcosine-PDK4 binding interface from molecular docking. **(D)** PDH activity in PDK4-KO and ΔAdom3 mutant cells ± sarcosine (0.5 mM, 48 h). **(E)** Western blot showing PDHA1 phosphorylation status in PDK4-KO and ΔAdom3 mutants. **(F-G)** Fluorescence images **(F)** and quantification **(G)** of ROS/ mitoROS in PDK4-KO cells pretreated with sarcosine (0.5 mM, 24 h), followed by RSL3 (2 µM) or IKE (10 µM, 8 h). Scale bars, 50 μm. **(H-I)** Dose-response curves of PDK4-KO cells exposed to RSL3 (A549:48 h; PC9:36 h) or IKE (A549:72 h; PC9:48 h; *n* = 4). Data were presented by mean (SD) and analyzed by one-way ANOVA with FDR adjustment. P-value less than 0.05 was considered as significant

Since sarcosine switched cell metabolism from glycolysis to oxidative phosphorylation, we speculated that sarcosine has an impact on the pyruvate dehydrogenase (PDH) activity. To this end, we employed a PDH activity kit and observed that sarcosine treatment, in contrast to its isomer alanine, resulted in an augmentation of PDH activity (Fig. 3A). Consistently, Western blot assays demonstrated that sarcosine treatment led to a reduction in PDHA1 phosphorylation at the S293 site (Fig. 3B). Molecular docking identified sarcosine as a putative interactor with the BCDHK_Adom3 domain of PDK4, a kinase responsible for PDHA1 inactivation (Fig. 3C). Subsequently, we constructed BCDHK_Adom3 domain-mutant (Asp 118, Asp 182, and Ser 183 were deleted) LUAD cells. Genetic ablation of PDK4 or targeted mutation of its BCDHK_Adom3 domain abolished sarcosine’s effects on PDH activity (Fig. 3D), PDHA1 phosphorylation status (Fig. 3E), and ROS generation (Fig. 3F & G). Importantly, PDK4 knockout or BCDHK_Adom3 domain-mutant cells exhibited heightened sensitivity to FINs (Fig. 3H & I), establishing PDK4 inhibition as the mechanistic link between sarcosine and ferroptosis potentiation. These findings delineate a previously unrecognized mechanism whereby sarcosine sensitizes LUAD cells to ferroptosis by directly targeting PDK4.

### Sarcosine activates NMDAR/MXD3 signaling to upregulate SLC40A1

**Fig. 4:**
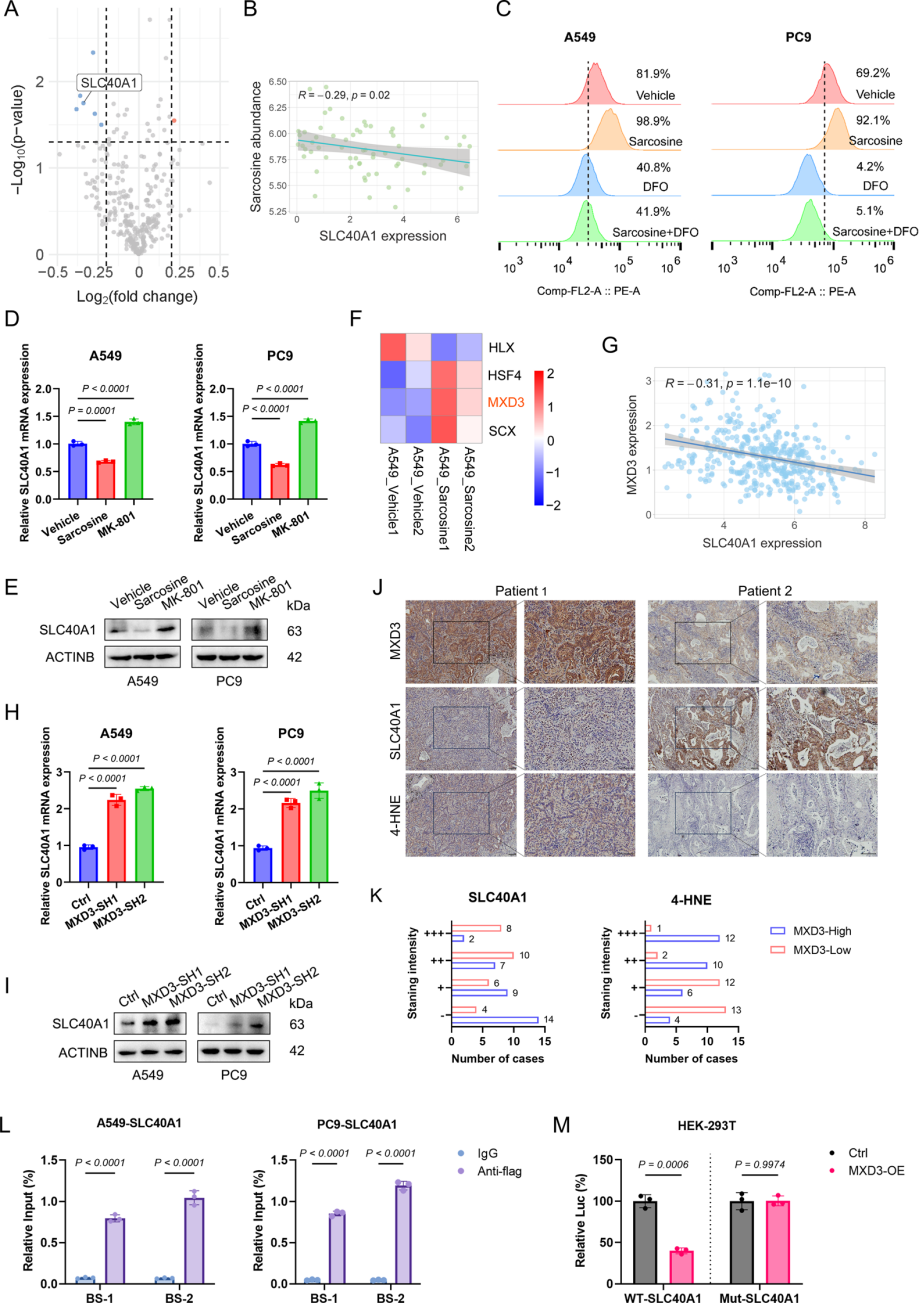
Sarcosine activates NMDAR/MXD3 signaling to upregulate SLC40A1. **(A)** Volcano plot of ferroptosis-related genes altered by sarcosine (0.5 mM, 48 h; red: up, blue: down). **(B)** The Spearman correlation analysis of SLC40A1 expression and sarcosine abundance in LUAD cells retrieved from the DEPMAP dataset. **(C)** Intracellular ferrous iron levels assessed via FerroOrange probes in LUAD cells pretreated with sarcosine (0.5 mM) and/or deferoxamine (DFO, 10 µM) for 24 h. **(D-E)** RT–qPCR (**D**) and Western blot (**E**) of SLC40A1 in cells treated with sarcosine (0.5 mM, 48 h) or MK-801 (10 µM, 48 h). **(F)** Heatmap of differentially expressed transcription factors (FC > 1.3 or < 0.7, FPKM > 2) after sarcosine treatment (0.5 mM, 48 h). **(G)** The Spearman correlation analysis of SLC40A1 and MXD3 expression in LUAD cells retrieved from the DEPMAP dataset. **(H-I)** RT–qPCR **(H)** and Western blot **(I)** of SLC40A1 after MXD3 knockdown. **(J-K)** IHC staining **(J)** and quantification **(K)** of MXD3, SLC40A1, and 4-HNE in LUAD specimens. Scale bars, 100 μm. **(L)** ChIP-qPCR showing MXD3 binding enrichment at SLC40A1 promoter regions. **(M)** Dual-luciferase reporter assays of wild-type vs. mutant SLC40A1 promoters. Data were presented by mean (SD) and analyzed by Student’s t-test or one-way ANOVA with FDR adjustment. P-value less than 0.05 was considered as significant

Sarcosine is reported to be a coactivator of N-methyl-D-aspartate receptor (NMDAR), we then explored the role of NMDAR on ferroptosis. Utilizing the NMDAR antagonist MK-801 and siRNAs targeting GRIN1, we elucidated the role of NMDAR in conferring ferroptotic sensitivity, as evidenced by the elevation of lipid peroxidation, intracellular ferrous ion levels, and MDA concentrations (Fig S4). Previous reports have found that NMDAR activation promotes iron deposition in cells. Notably, we identified SLC40A1, a critical transmembrane protein involved in ferrous ion export, as one of the most prominently altered genes upon sarcosine treatment (Fig. 4A). To further validate these observations, we harnessed multi-omics data from the DEPMAP dataset, revealing a negative correlation between SLC40A1 expression and sarcosine abundance in LUAD cells (Fig. 4B). The FerroOrange probes demonstrated that sarcosine treatment elevated intracellular ferrous iron levels, which can be reversed by DFO (Fig. 4C). Subsequently, we experimentally confirmed that sarcosine negatively regulates SLC40A1 expression, whereas MK-801 treatment elicited an opposing effect (Fig. 4D & E).

To dissect the transcriptional regulation of SLC40A1, we analyzed the transcriptomic profile of A549 cells treated with sarcosine, pinpointing HLX, HSF4, MXD3, and SCX as the most prominently altered transcription factors (Fig. 4F). Given the minimal variation observed in HLX, HSF4, and SCX expression in PC9 cells, we focused our attention on MXD3 (Fig S5A). Our RNA-seq analysis of A549 and PC9 cells following FIN treatment indicated a robust downregulation of MXD3, suggesting its potential pro-ferroptotic function in LUAD (Fig S5B & C). Moreover, transcriptomic interrogation of LUAD cell lines from the DEPMAP dataset revealed an inverse relationship between MXD3 and SLC40A1 expression (Fig. 4G). To experimentally validate the pro-ferroptotic role of MXD3, we generated LUAD cell lines with MXD3 knockdown or overexpression (Fig S5D & E) and employed a series of functional assays, which firmly established the pro-ferroptotic effect of MXD3 in LUAD (Fig S5F - J). Subsequently, we confirmed that MXD3 knockdown led to a robust upregulation of SLC40A (Fig. 4H & I). IHC staining also revealed an inverse correlation between MXD3 and SLC40A1 expression, as well as a positive correlation between MXD3 and 4-HNE, a biomarker of lipid peroxidation (Fig. 4J & K).

ChIP-seq data from the ENCODE database (https://www.encodeproject.org/) identified MXD3 binding sites within the SLC40A1 promoter, which were validated by ChIP-qPCR (Fig. 4L, S5K-L). Luciferase reporter assays demonstrated that MXD3 directly represses SLC40A1 transcription, as mutations in the predicted binding motifs abolished this effect (Fig. 4M, S5M-N). Besides, genetic ablation of SLC40A1 sensitized LUAD cells to FINs and abrogated the ferroptosis-promoting effects of MXD3 and MK-801, underscoring SLC40A1 as a critical downstream effector of sarcosine (Fig S6).

Collectively, we delineated a novel NMDAR/MXD3/SLC40A1 axis through which sarcosine potentiates ferroptosis in LUAD. By activating NMDAR, sarcosine induces MXD3-mediated transcriptional repression of SLC40A1, leading to intracellular iron accumulation.

### PDK4 activation and NMDAR Blockade reverse sarcosine-mediated ferroptosis

**Fig. 5:**
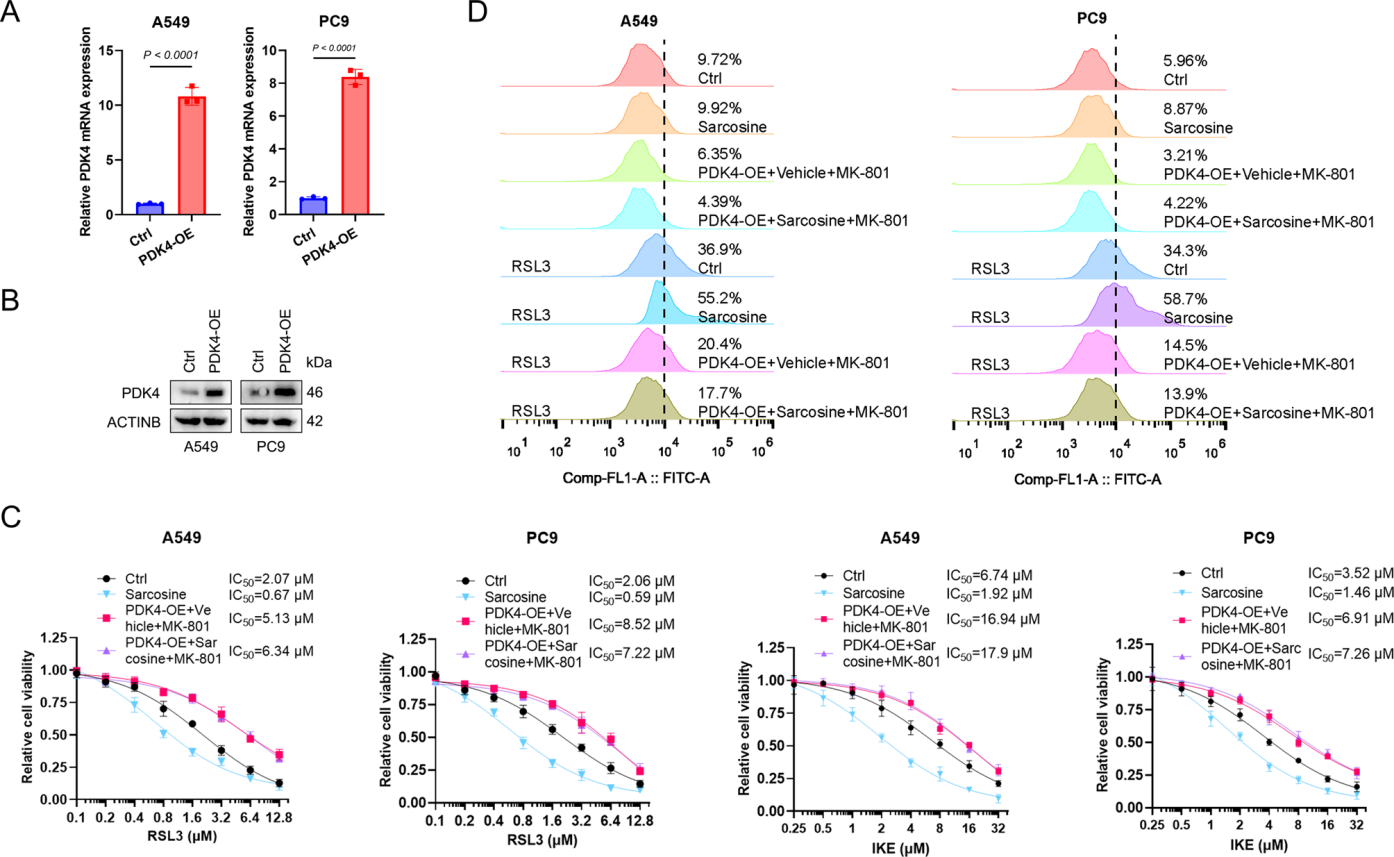
PDK4 activation and NMDAR blockade reverse sarcosine-mediated ferroptosis. **(A-B)** PDK4 overexpression validation in LUAD cells by RT–qPCR (**A**) and Western blot (**B**). **(C)** Dose-response curves of LUAD cells treated with RSL3 (A549:48 h; PC9:36 h) or IKE (A549:72 h; PC9:48 h) ± MK-801 (10 µM). **(D)** Lipid-ROS levels in cells pretreated with MK-801 (10 µM, 24 h) ± RSL3 (2 µM, 8 h). Data were presented by mean (SD) and analyzed by Student’s t-test. P-value less than 0.05 was considered as significant

This study elucidates the mechanistic basis of ferroptosis, highlighting the roles of PDK4-mediated ROS generation and the activation of the NMDAR/MXD3/SLC40A1 signaling axis. To investigate the regulatory effects of PDK4 and NMDAR on sarcosine-induced ferroptosis, we employed LUAD cell lines engineered to overexpress PDK4 (Fig. 5A & B). Our results demonstrated that the combined ectopic expression of PDK4 and pharmacological inhibition of NMDAR significantly attenuates the pro-ferroptotic effects of sarcosine (Fig. 5C & D), underscoring the effect of PDK4 and NMDAR in modulating ferroptosis.

### Sarcosine enhances cisplatin (CDDP) sensitivity through ferroptosis

**Fig. 6:**
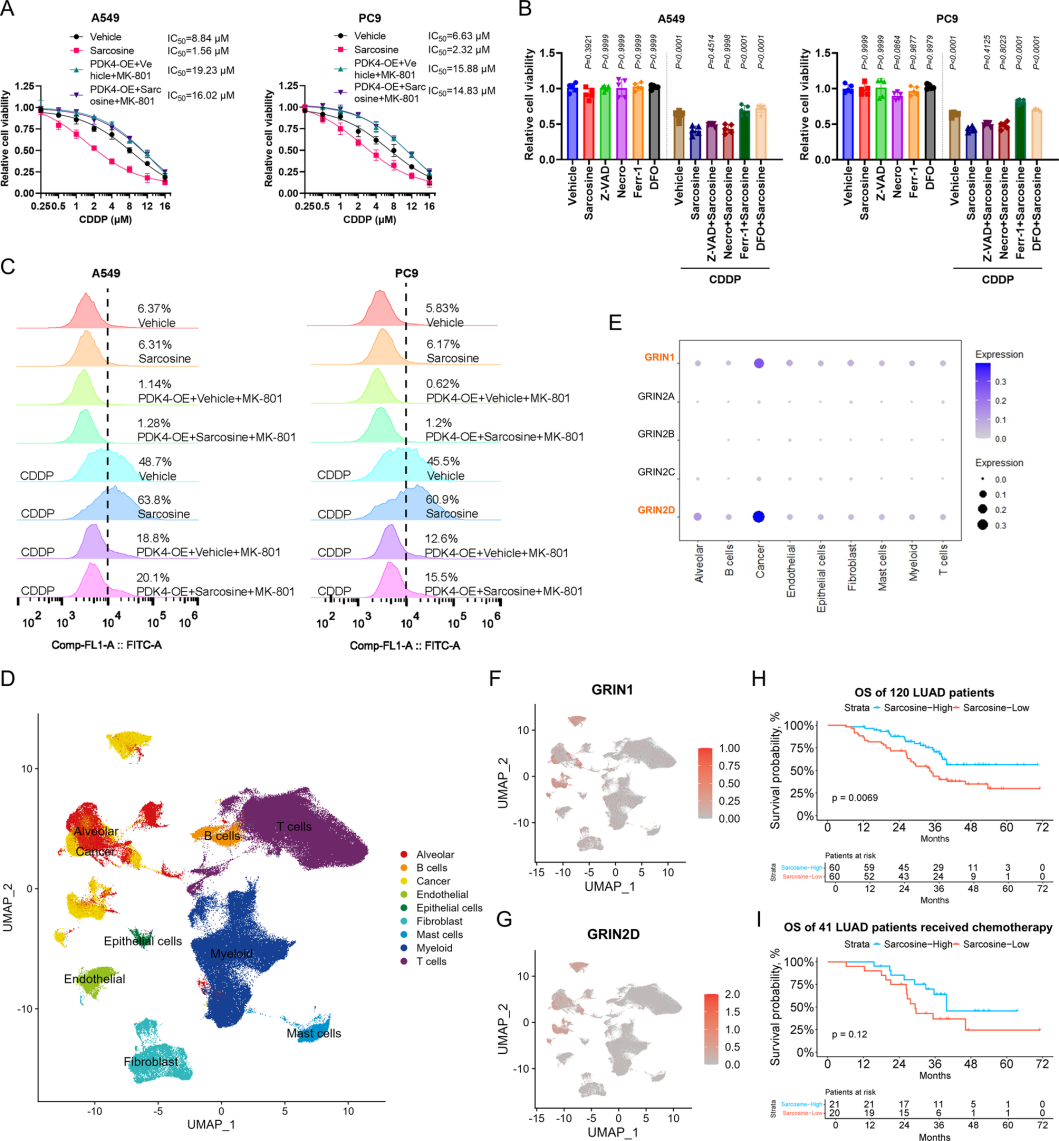
Sarcosine enhances cisplatin (CDDP) sensitivity through ferroptosis. **(A)** Viability of LUAD cells treated with CDDP (72 h) ± MK-801 (10 µM)/sarcosine (0.5 mM; *n* = 5). **(B)** Rescue effects of cell death inhibitors (10 µM each) on sarcosine-potentiated cytotoxicity. **(C)** Lipid-ROS levels in CDDP-treated (10 µM, 24 h) cells ± MK-801/sarcosine. **(D)** UMAP projection of single-cell transcriptomes from 12 normal and 17 LUAD tissues. **(E-G)** Cell-type-specific expression of NMDAR subunits in tumor ecosystems. **(H-I)** Kaplan-Meier survival curves stratified by tumor sarcosine levels: overall cohort (**H**, *n* = 120) and CDDP-treated subgroup (**I**, *n* = 41). Data were presented by mean (SD) and analyzed by one-way ANOVA with FDR adjustment. P-value less than 0.05 was considered as significant

Platinum-based chemotherapy alone or in combination with other therapies has been established as an effective modality for LUAD patients. Previous reports have uncovered the pivotal role of ferroptosis in CDDP-induced cell death [22]. Given these findings, we sought to explore the potential impact of sarcosine on chemosensitivity in LUAD cells. Our results indicated that exogenous supplementation of sarcosine sensitized LUAD cells to CDDP treatment, and the chemo-sensitizing effect was substantially attenuated by the ferroptosis inhibitors Fer-1 and DFO (Fig. 6A-C).

To further substantiate our findings, we leveraged single-cell sequencing data of 12 normal lung and 17 LUAD tissue samples from our previously published work [21]. The results demonstrated a preferential expression pattern of GRIN1 and GRIN2D (the encoding genes of NMDAR) in tumor cells, highlighting the tumor-specific properties of sarcosine (Fig. 6D - G). Additionally, we examined resected tumor tissues and clinical data from 120 LUAD patients, including 41 who received CDDP-based chemotherapy. Patients were stratified into high- and low-sarcosine groups based on median sarcosine levels measured by a sarcosine assay kit. Kaplan-Meier survival analysis demonstrated a significant association between elevated sarcosine levels and improved patient outcomes (Fig. 6H & I, Supplementary Tables 2 & 3). Consistent with these findings, serum sarcosine levels in 100 LUAD patients also correlated positively with survival, further supporting the prognostic relevance of sarcosine (Fig S7, Supplementary Table 4). Collectively, these data underscore the therapeutic potential of sarcosine in augmenting CDDP efficacy and improving clinical outcomes in LUAD patients.

### Sarcosine potentiates therapeutic efficacy in patient-derived organoids (PDOs) and xenografts

**Fig. 7:**
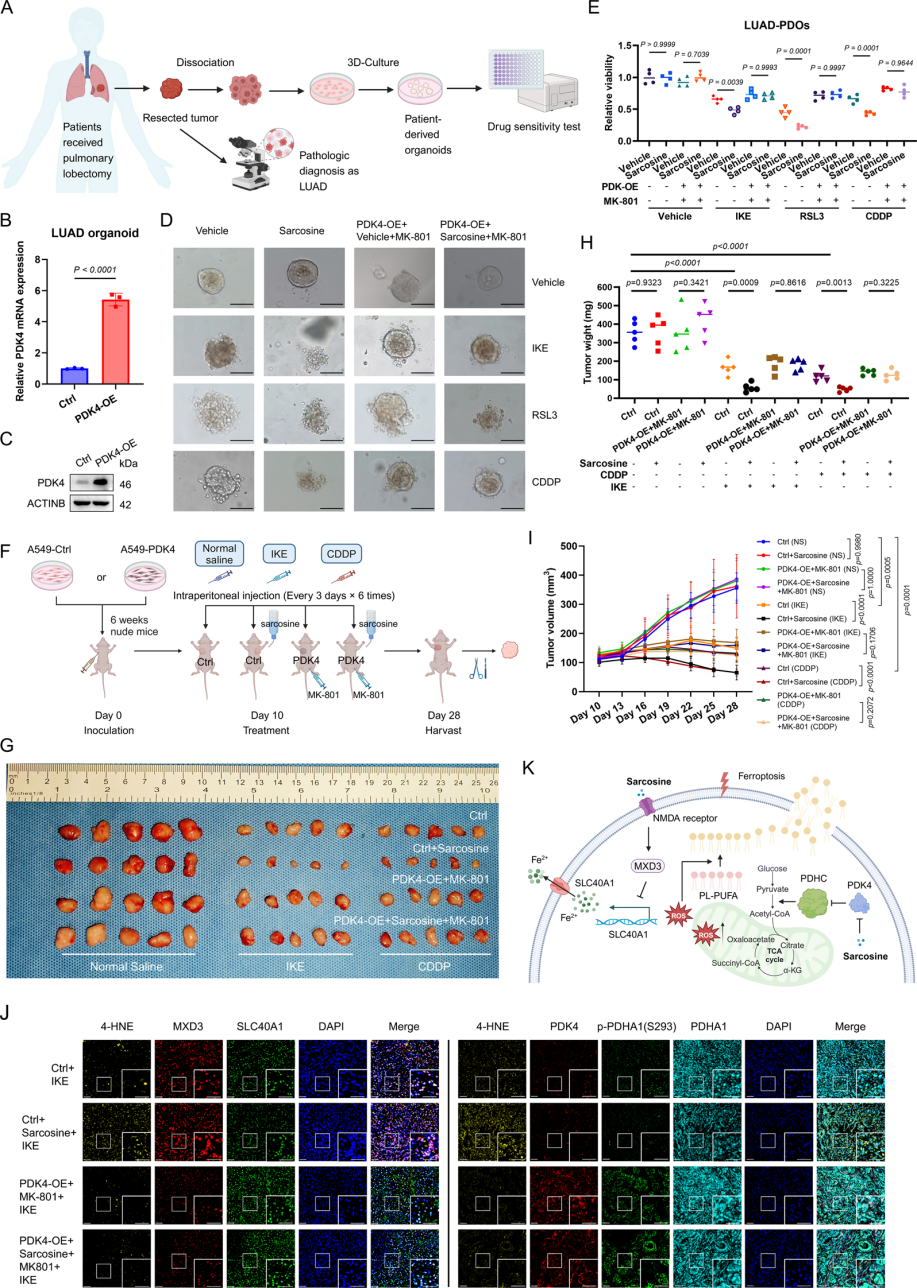
Sarcosine potentiates therapeutic efficacy in patient-derived organoids (PDOs) and xenografts. **(A)** Workflow for PDO establishment. **(B-C)** PDK4 overexpression validation in PDOs by RT–qPCR (**B**) and Western blot (**C**). **(D-E)** Brightfield images (**D**, scale bars 100 μm) and viability of PDOs (**E**) treated with IKE (20 µM), RSL3 (10 µM), or cisplatin (30 µM) ± sarcosine (0.5 mM)/MK-801 (10 µM) for 120 h. **(F)** Experimental design for subcutaneous xenograft models (*n* = 5/group). **(G-I)** Tumor specimens **(G)**, weights **(H)**, and growth curves **(I)** across treatment groups. **(J)** Multiplex IHC of 4-HNE and target proteins in tumor sections. Scale bars, 50 μm. **(K)** Mechanistic schema of sarcosine-regulated ferroptosis in LUAD. Data were presented by mean (SD) and analyzed by Student’s t-test, one-way otr two-way ANOVA with FDR adjustment. P-value less than 0.05 was considered as significant

PDOs exhibited a remarkable capacity to imitate the histological and molecular hallmarks of their primary tissues, thereby providing an ideal platform for drug sensitivity tests [23, 24]. After constructing PDK4-OE PDOs via lentivirus transfection, we found that sarcosine supplementation sensitized PDOs to FINs and CDDP through morphological changes and ATPase activity measurement (Fig. 7A - C). What’s more, this sensitizing effect was abolished in PDK4-OE PDOs treated with MK-801 at the same time (Fig. 7D & E).

To extend these findings in vivo, we established xenograft models using Ctrl and PDK4-overexpressing A549 cells, with IKE as the FIN due to its stability in animals [25] (Fig. 7F). Strikingly, IKE and CDDP treatment significantly inhibited tumor growth, and this effect was further amplified by sarcosine supplementation in Ctrl tumors, yet not in PDK-OE tumors administered with MK-801 (Fig. 7G - I). Multiplex IHC analysis of the IKE-treated group revealed that sarcosine treatment contributed to ferroptosis, which can be rescued by PDK4 and MK-801 (Fig. 7J). These findings demonstrated that sarcosine enhances ferroptosis and chemotherapy sensitivity through a mechanism dependent on PDK4 inhibition and NMDAR activation, which highlights the therapeutic potential of sarcosine to overcome resistance to ferroptosis-inducing therapies in LUAD.

## Discussion

Ferroptosis, an iron-dependent cell death process driven by lipid peroxidation, represents a therapeutic vulnerability in cancers resistant to other cell death pathways [26]. Preclinical studies further support the synergistic potential of combining FINs with existing cancer treatments [5, 27, 28]. Herein, we identified sarcosine as a critical ferroptosis amplifier in LUAD using two mechanistically distinct FINs, RSL3 (GPX4 inhibitor) and IKE (a stabilized derivative of erastin that suppresses the cystine/glutamate antiporter). Mechanistic studies revealed that sarcosine potentiates ferroptosis by shifting cellular metabolism from glycolysis to oxidative phosphorylation and promoting iron retention through the NMDAR/MXD3/SLC40A1 axis. These effects synergize with cisplatin to enhance chemosensitivity, establishing sarcosine as a vulnerability for ferroptosis-based cancer therapy (Fig. 7K).

Sarcosine, also known as N-methyl glycine, is an endogenous amino acid that is a competitive inhibitor of the type I glycine transporter, an NMDAR co-agonist, and an important intermediate in one-carbon metabolism [29]. In the context of prostate cancer, sarcosine was identified as a pivotal differential metabolite that exhibited a pronounced upregulation during the tumor progression toward metastatic stages [16]. Khan et al. further observed a significant upregulation of the sarcosine biosynthetic enzyme, GNMT, in tumor tissues, while the expression of enzymes involved in sarcosine metabolism, namely SARDH and PIPOX, is notably downregulated. Besides, exogenous sarcosine triggers invasion and intravasation in an in-vivo prostate cancer model [13].

Beyond prostate cancer, sarcosine has also been implicated in other malignancies. For instance, the plasma sarcosine profile demonstrates the potential to distinguish colorectal cancer patients from those with precancerous conditions and healthy individuals [30]. In breast cancer, the expression of proteins related to sarcosine metabolism has been associated with patient prognosis [31]. Moreover, urinary sarcosine levels have been explored as a diagnostic tool for hepatocellular carcinoma. A chemical biopsy of urinary sarcosine could facilitate early and noninvasive diagnosis of this disease, with direct implications for tumorigenesis [32]. Additionally, Dastmalchi et al. demonstrated that sarcosine enhances the migratory capacity of murine and human dendritic cells through the CXC chemokine pathway, leading to a more robust anti-tumor immune response and improved tumor control in animal models [33]. However, little research focused on the role of sarcosine in ferroptosis and LUAD, while our metabolic library screening results identified sarcosine as a potent inducer of ferroptosis in LUAD for the first time.

Untargeted metabolomic profiling of LUAD cells revealed a distinct metabolic signature marked by sarcosine downregulation after RSL3 treatment. Notably, cells exhibiting elevated sarcosine levels displayed heightened sensitivity to RSL3-mediated cytotoxicity, whereas cells that survived such treatment tended to have lower sarcosine concentrations. Next, we observed minimal metabolic transformation of sarcosine in LUAD cells through isotope tracing. The untargeted metabolomic profiling and seahorse assays unveiled a critical metabolic nexus linking sarcosine-induced ferroptosis to PDH enzyme activation in LUAD. The mitochondrial PDH serves as an indispensable interface between glycolysis and the TCA cycle, mediating the decarboxylation of pyruvate to acetyl coenzyme A [34]. Besides immediate substrates and products, the regulation of the PDH is primarily achieved through covalent modifications of the E1α subunit (that is PDHA1), the rate-limiting enzyme within the complex [35]. Among these modifications, reversible phosphorylation of E1α stands out as the most extensively studied mechanism. Specifically, phosphorylation by PDK at any one of three serine residues on E1α (site 1, Ser-293; site 2, Ser-300; site 3, Ser-232) inhibits PDH activity, among which Ser-293 is the most rapidly regulated site [36]. By suppressing PDK4, sarcosine alleviates PDHA1 phosphorylation at Ser293, unleashing PDH activity to redirect glycolytic flux into the TCA cycle—a metabolic rewiring that amplifies mitochondrial ROS and drives ferroptosis. This PDH-centric mechanism explains sarcosine’s ability to override the Warburg effect, forcing LUAD cells into oxidative phosphorylation.

Sarcosine is reported to be a coactivator of NMDAR, which are ligand-gated voltage-dependent channels belonging to the ionotropic glutamate receptor family and play an important role in synaptic activities as well as mental disorders [37, 38]. In this study, we demonstrated that NMDAR activation, by targeting the MXD3/SLC40A1 axis, can effectively augment LUAD cell ferroptosis via inhibiting ferrous export. Similarly, Wang et al. reported that NMDAR activation exacerbates iron uptake to promote ferroptosis in intracerebral hemorrhage [39]. Notably, recent studies implicate NMDAR signaling in macrophage-driven tumor progression [40, 41], yet sarcosine’s role in immune-tumor microenvironment crosstalk remains unclear. While our data highlight sarcosine’s direct antitumor activity in T-cell-deficient models, future studies in immunocompetent systems are warranted to elucidate its potential synergy with immune surveillance mechanisms.

Chemotherapy continues to occupy a pivotal position in cancer management, yet its efficacy is challenged by the emergence of chemoresistance [42]. Amidst this challenge, mounting studies have highlighted the role of ferroptosis-inducing strategies to overcome chemoresistance [7, 43]. In cisplatin-resistant non-small cell lung cancer, the utilization of the NQO1 substrate, 2-methoxy-6-acetyl-7-methyljuglone, induced ferroptosis and offered a potential avenue to circumvent chemoresistance [44]. In head and neck squamous cell carcinoma, the implementation of FINs targeting SLC7A11 presents a promising approach to alleviate cisplatin resistance [45]. Likewise, we found that sarcosine sensitizes LUAD to cisplatin via ferroptosis, indicating that sarcosine supplementation is a feasible strategy to augment cisplatin-based chemotherapeutic efficacy.

The intersection between ferroptosis and dietary interventions in cancer therapy has gained considerable interest. Preclinical studies have demonstrated that dietary supplementation with arachidonic acid can augment antitumor immunity, while dietary restriction of methionine and cysteine has the potential to sensitize solid tumors to ferroptosis [46, 47]. Notably, sarcosine exhibits no discernible toxicity, as underscored by the absence of phenotypic manifestations associated with sarcosinemia, a congenital disorder arising from aberrant sarcosine metabolism [48]. Furthermore, oral supplementation with sarcosine has been used in the treatment of depression and schizophrenia [49, 50]. However, its mechanism remains unclear whether effects arise from central penetration through brain-blood barrier or peripheral metabolic pathways. While these findings underscore diet-driven ferroptosis regulation as a viable cancer treatment strategy, critical gaps persist in understanding sarcosine’s metabolic fate and transport dynamics following dietary intervention, necessitating systematic pharmacokinetic investigations.

## Conclusion

Collectively, we discovered that sarcosine enhances ferroptosis by inhibiting PDK4 to boost ROS and activating the NMDAR/MXD3/SLC40A1 axis to promote iron retention. Clinically, sarcosine synergizes with cisplatin, correlating with improved survival in patients. With a favorable safety profile, sarcosine emerges as a promising adjuvant to amplify platinum-based therapy efficacy in LUAD.

## Electronic supplementary material

Below is the link to the electronic supplementary material.

Supplementary Material 1

Supplementary Material 2

Supplementary Material 3

## Data Availability

The datasets used and analyzed during the current study are available from the corresponding author on reasonable request.

## Abbreviations

LUAD: Lung Adenocarcinoma
ROS: Reactive Oxygen Species
H_2_O_2_: Hydrogen Peroxide
TCA cycle: Tricarboxylic Acid Cycle
FINs: Ferroptosis Inducers
IKE: Imidazole Ketone Erastin
RSL3: RAS-Selective Lethal 3
Necro: Necrosulfonamide
Fer-1: Ferrostatin-1
DFO: Deferoxamine
CDDP: Cisplatin
MDA: Malondialdehyde
IHC: Immunohistochemistry
PDH: Pyruvate Dehydrogenase
ChIP: Chromatin Immunoprecipitation
NMDAR: N-Methyl-D-Aspartate Receptor
PDOs: Patient-Derived Organoids
