# Supplementary material for: Sarcosine sensitizes lung adenocarcinoma to chemotherapy by dual activation of ferroptosis via PDK4/PDHA1 signaling and NMDAR-mediated iron export
Source: Exp Hematol Oncol. 2025 Apr 24;14:60. doi: 10.1186/s40164-025-00657-0 (PMC12023509; doi:10.1186/s40164-025-00657-0)
Supplement: Supplementary file 2 — Supplementary Material 2 [file 40164_2025_657_MOESM2_ESM.docx]

**
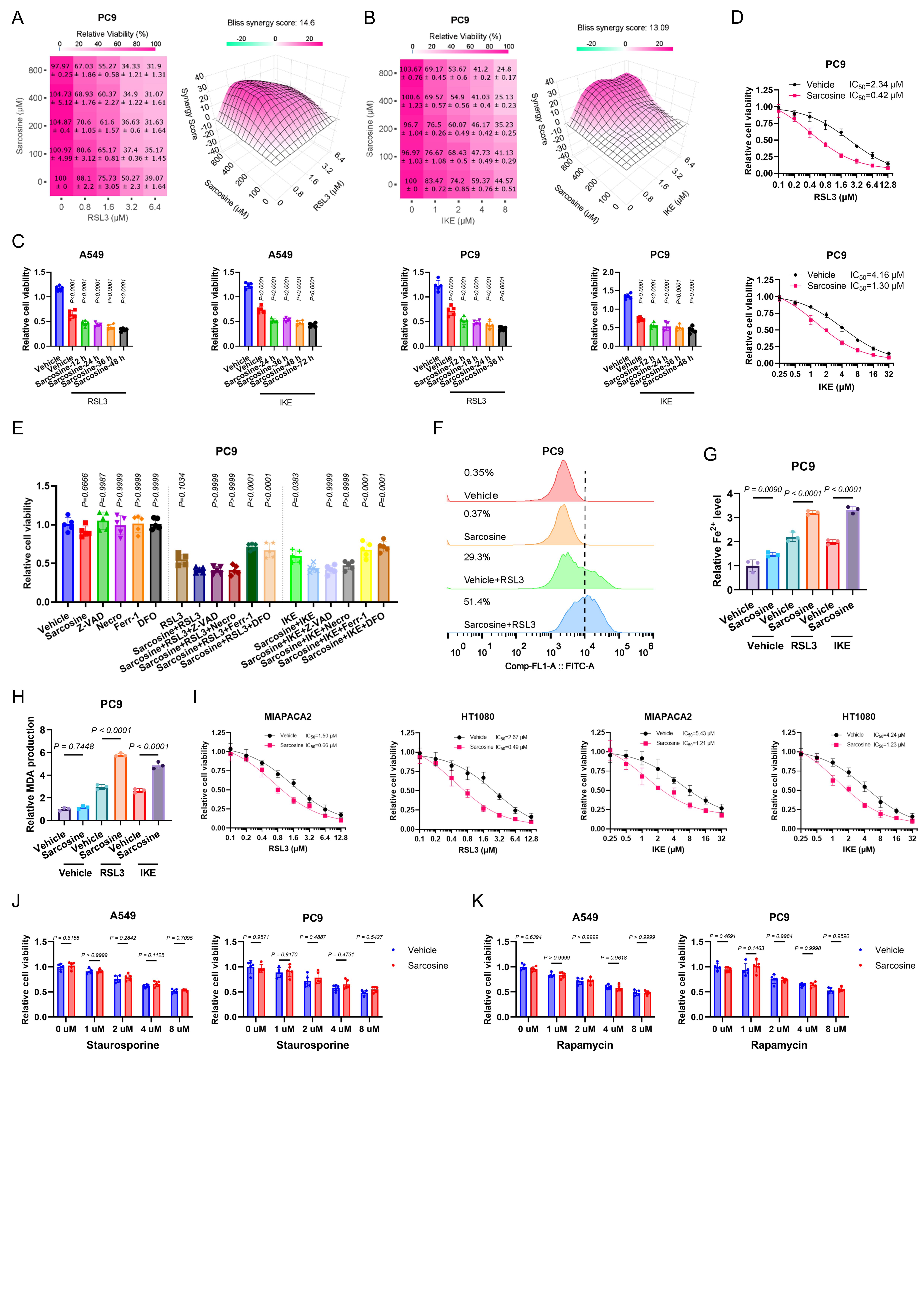
Fig S1**

**Figure S1 Sarcosine synergizes with ferroptosis inducers (FINs) across cell models.**
**(A-B)** Bliss synergy scores in PC9 cells treated with RSL3 (36 h) or IKE (48 h). **(C)** Viability of LUAD cells exposed to sarcosine (0.5 mM) ± RSL3/IKE (A549:48/72 h; PC9:36/48 h) (n=4). **(D)** Dose-response curves of sarcosine-co-treated (0.5 mM) PC9 cells (n=4). **(E)** Rescue effects of cell death inhibitors (10 μM each) on sarcosine-enhanced cytotoxicity. **(F-H)** Lipid-reactive oxygen species, ferrous iron, and malondialdehyde (MDA) levels in PC9 cells pretreated with sarcosine (0.5 mM, 24 h) followed by RSL3 (2 μM) or IKE (10 μM, 8 h). **(I)** Viability of MIAPACA2/HT1080 cells treated with RSL3 (6/72 h) or IKE (12/72 h) ± sarcosine (0.5 mM, n=5). **(I-K)** Viability of LUAD cells treated with apoptosis inducer staurosporine or autophagy inducer Rapamycin ± sarcosine (0.5 mM, n=5). Data were presented by mean (SD) and analyzed by one-way analysis of variance (ANOVA) with FDR adjustment. P-value less than 0.05 was considered as significant.


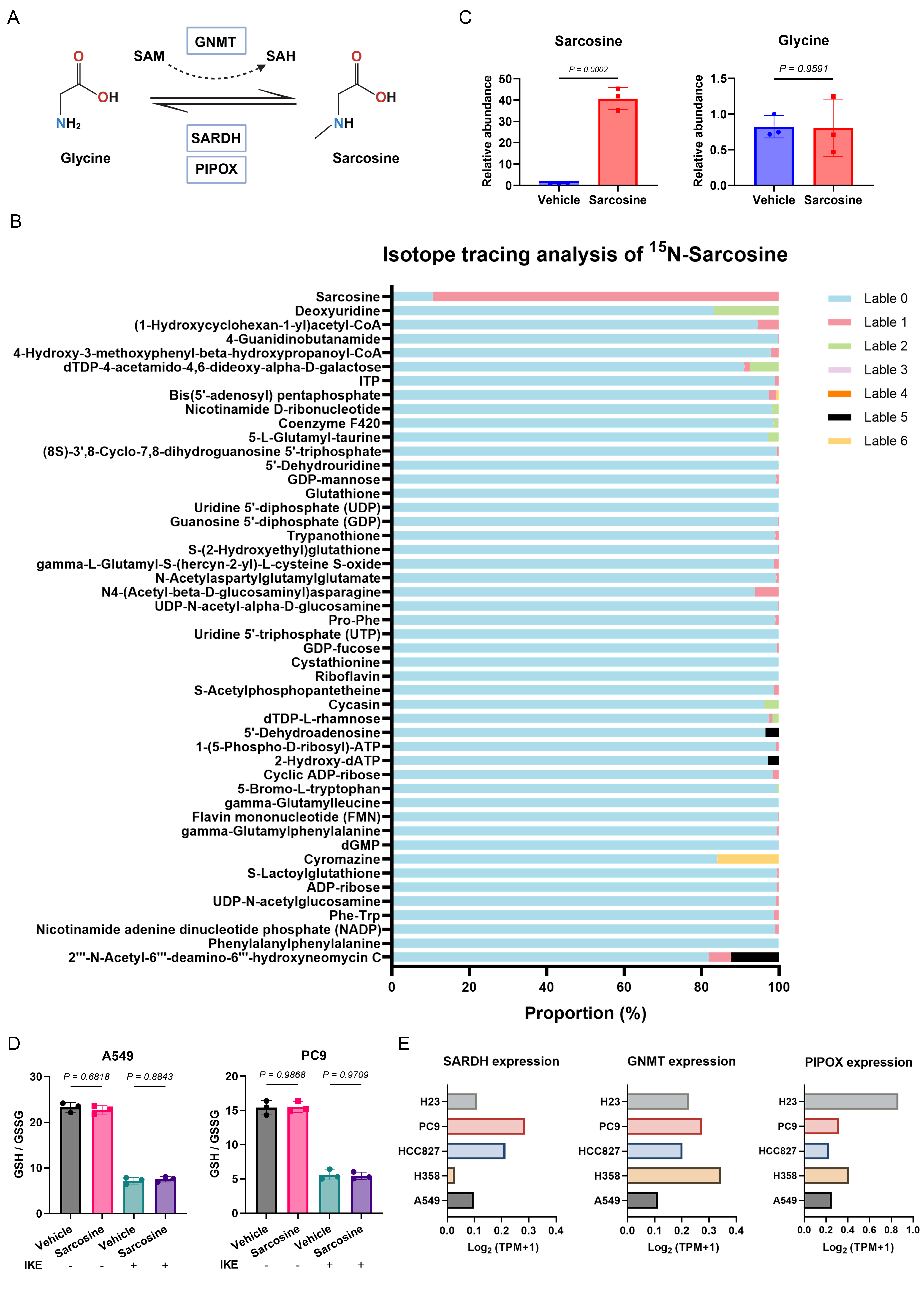
**Fig S2**

**Figure S2 Sarcosine undergoes minimal conversion to other metabolites in LUAD. (A)** Enzymatic network mediating sarcosine-glycerate conversion (SARDH/PIPOX/GNMT). **(B)** The labeled metabolites identified by the metabolic flux analysis. A549 cells were incubated with ^15^N-sarcosine (0.5 mM) for 48 h. **(C)** Sarcosine/glycine abundance in sarcosine-supplemented cells (0.5 mM, 48 h). **(D)** GSH/GSSG ratio in cells treated with sarcosine (0.5 mM, 48 h) ± IKE (2 μM, 8 h). **(E)** Expression profiles (log2[TPM+1]) of sarcosine-metabolizing enzymes in LUAD cell lines obtained from the DEPMAP dataset. Data were presented by mean (SD) and analyzed by Student’s t-test or one-way analysis of variance (ANOVA) with FDR adjustment. P-value less than 0.05 was considered as significant.

**
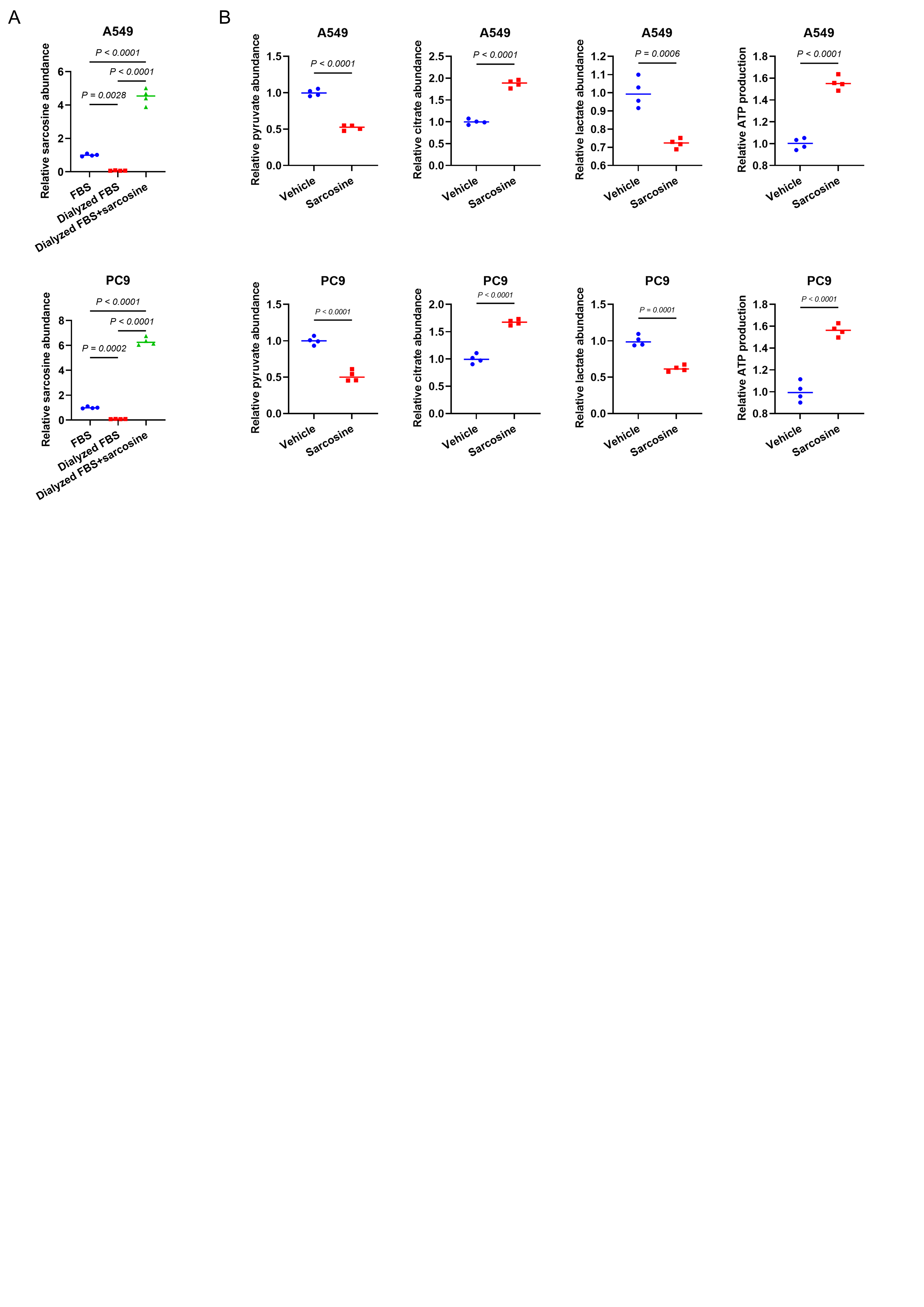
Fig S3**

**Figure S3 Sarcosine modulates cellular energy metabolism.**
**(A)** Sarcosine levels in cells cultured with 10% standard/dialyzed FBS ± sarcosine (0.5 mM, 48 h). **(B)** Relative pyruvate, citrate, lactate, and ATP levels in sarcosine-treated cells (0.5 mM, 48 h).  Data were presented by mean (SD) and analyzed by Student’s t-test or one-way analysis of variance (ANOVA) with FDR adjustment. P-value less than 0.05 was considered as significant.

**
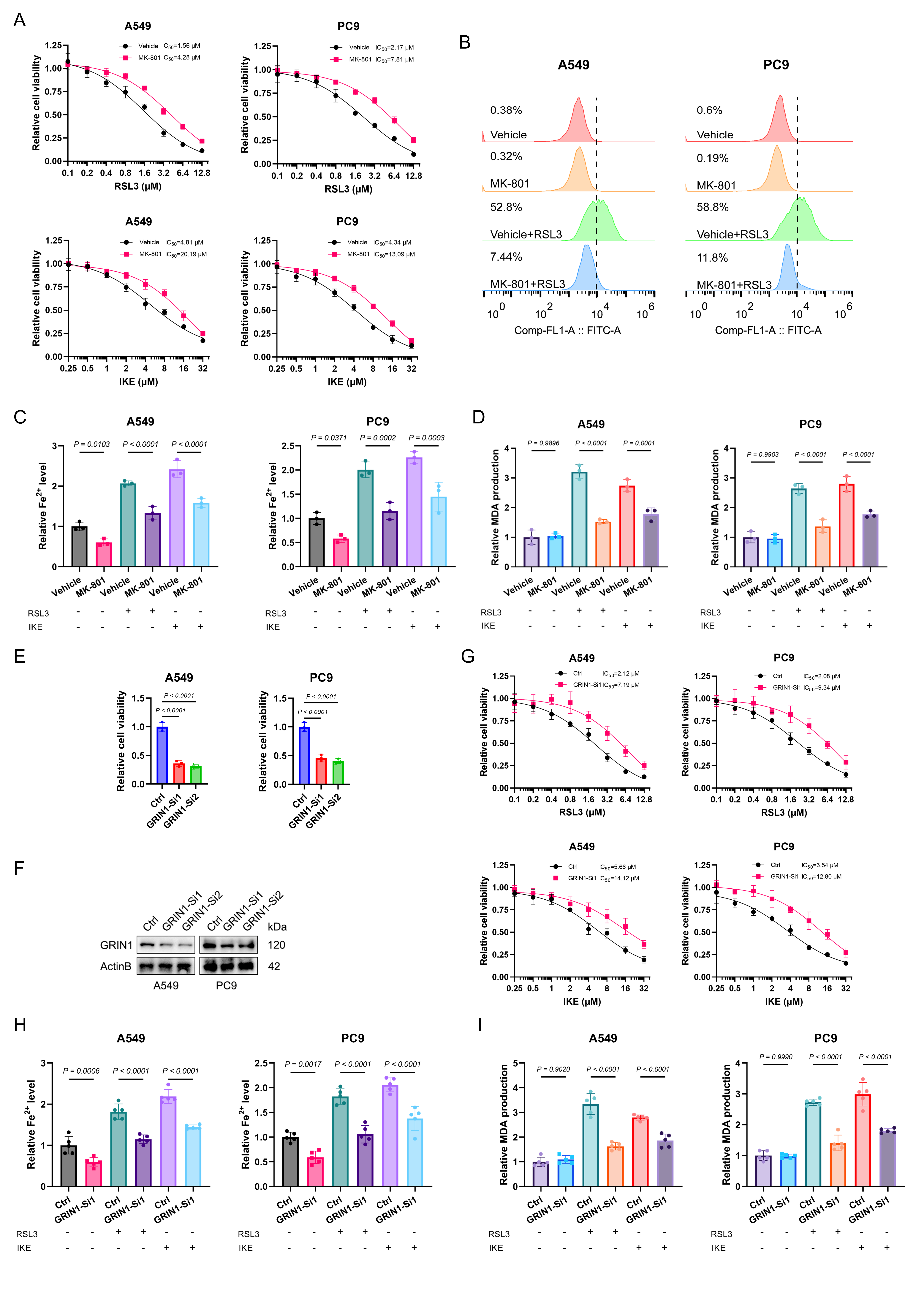
Fig S4**

**Figure S4 NMDAR blockade blunts ferroptosis in LUAD.**
**(A)** Viability of MK-801-pretreated (10 μM, 24 h) LUAD cells exposed to RSL3 (A549:48 h; PC9:36 h) /IKE (A549:72 h; PC9:48 h). **(B-D)** Lipid-ROS, ferrous iron, and MDA levels in MK-801-treated cells ± RSL3 (2 μM, 8 h)/IKE (10 μM, 8 h). **(E-F)** GRIN1 knockdown validation by qPCR/Western blot. **(G-I)** Ferroptosis susceptibility in GRIN1-KD cells: cell viability (G), ferrous iron (H), and MDA (I). Data were presented by mean (SD) and analyzed by one-way analysis of variance (ANOVA) with FDR adjustment. P-value less than 0.05 was considered as significant.

**
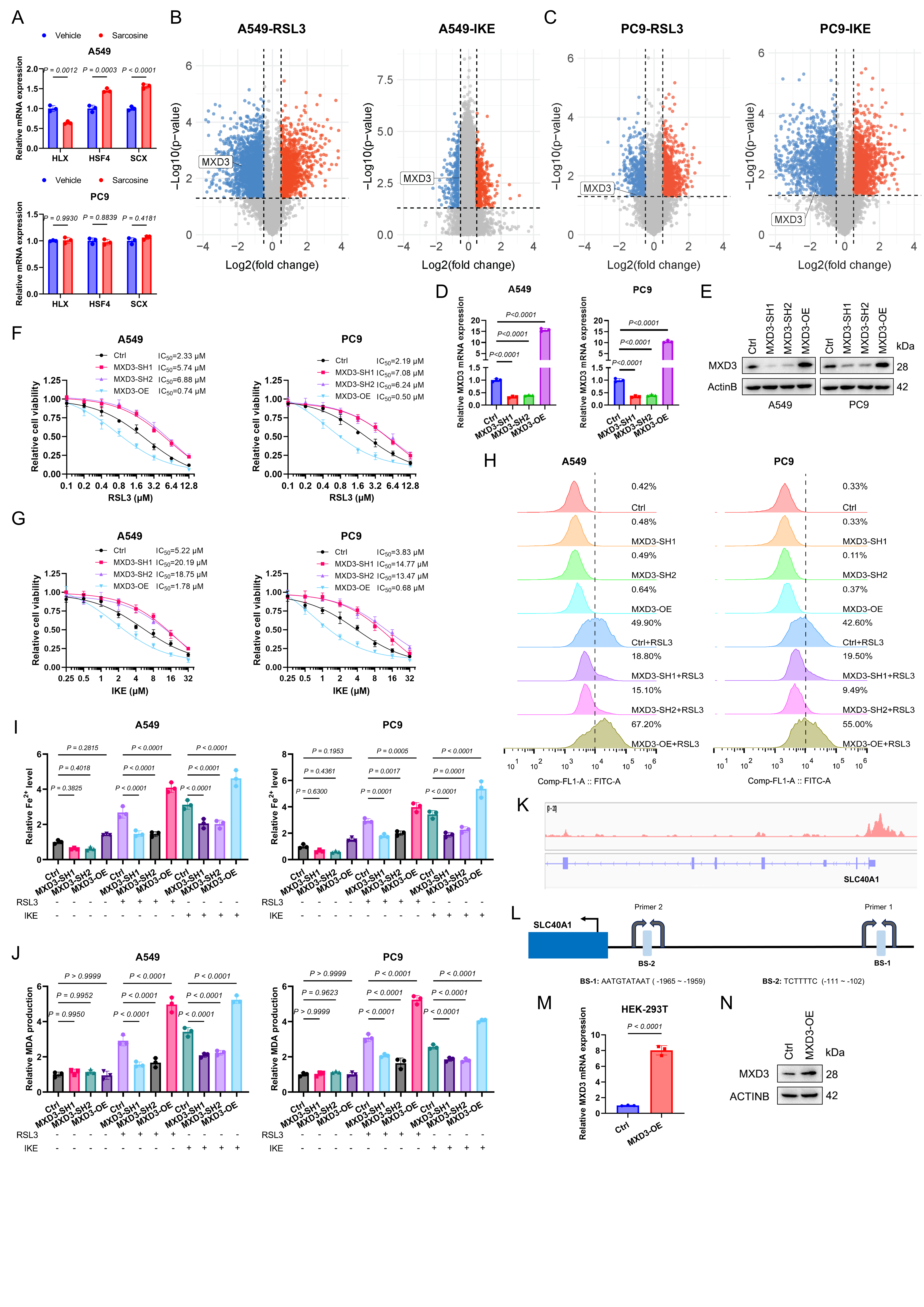
Fig S5**

**Figure S5 MXD3 promotes ferroptosis in LUAD.**
**(A)** HLX/HSF4/SCX mRNA expression in sarcosine-treated cells (0.5 mM, 48 h). **(B-C)** Differentially expressed genes in RSL3 (5 μM, 24 h)/IKE-treated (10 μM, 48 h) vs control cells. **(D-E)** MXD3 knockdown/overexpression validation by qPCR/Western blot. **(F-J)** Ferroptosis phenotypes in modified cells: cell viability (F), lipid-ROS (H), ferrous iron (I), MDA (J). **(K-L)** ChIP-seq peaks (K) and predicted MXD3 binding sites (L) at SLC40A1 promoter. **(M-N)** MXD3 overexpression in HEK-293T cells. Data were presented by mean (SD) and analyzed by Student’s t-test or one-way analysis of variance (ANOVA) with FDR adjustment. P-value less than 0.05 was considered as significant.

**
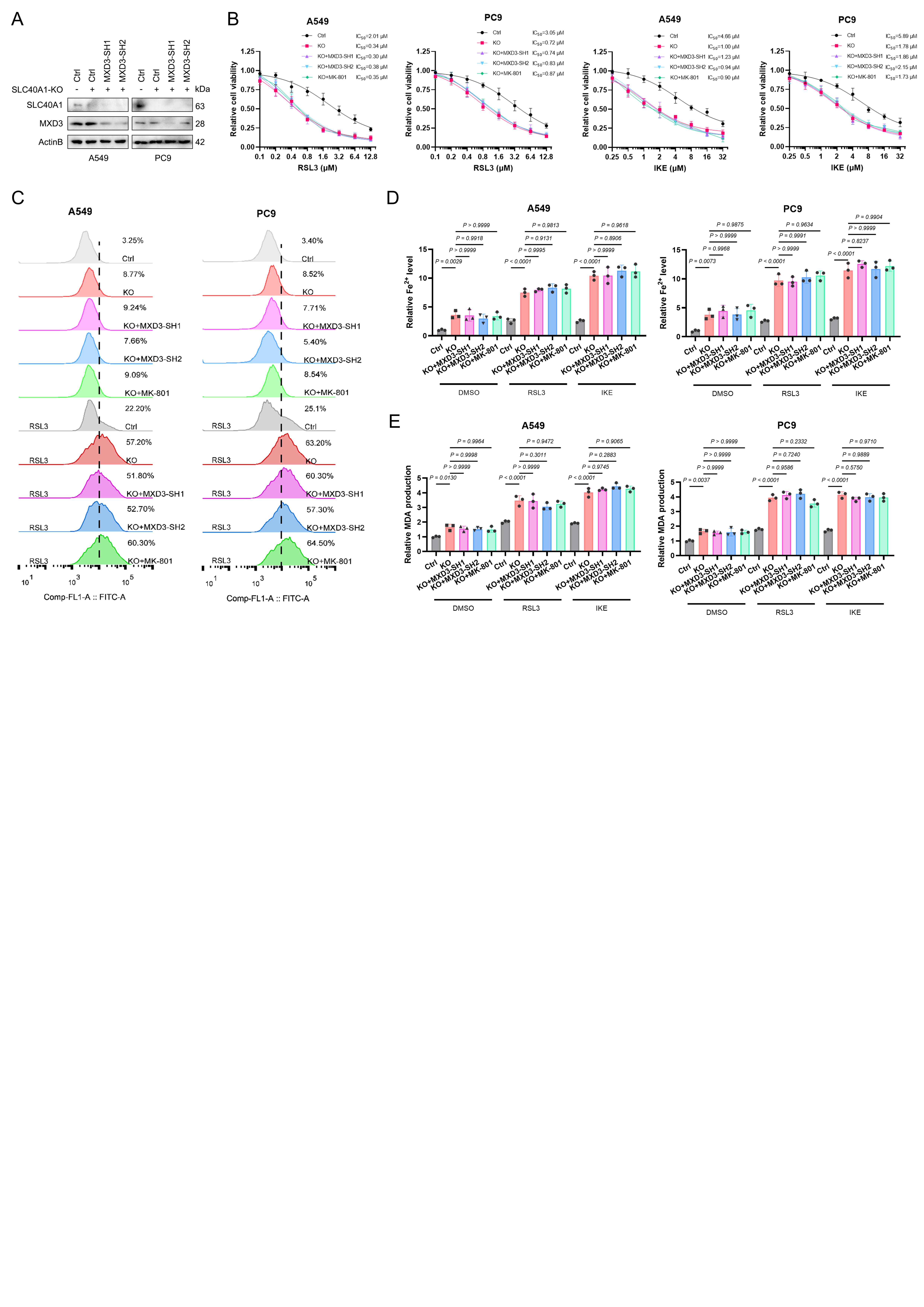
Fig S6**

**Figure S6 SLC40A1 knockout confers ferroptosis sensitivity in LUAD.**
**(A)** SLC40A1 knockout validation by Western blot. **(B)** Viability of SLC40A1-KO cells ± MK-801 pretreatment (10 μM, 24 h). **(C-E)** Lipid-ROS, ferrous iron, and MDA levels in SLC40A1-KO cells under RSL3 (4 μM, 8 h)/IKE (10 μM, 8 h) treatment. Data were presented by mean (SD) and analyzed by one-way analysis of variance (ANOVA) with FDR adjustment. P-value less than 0.05 was considered as significant.

**
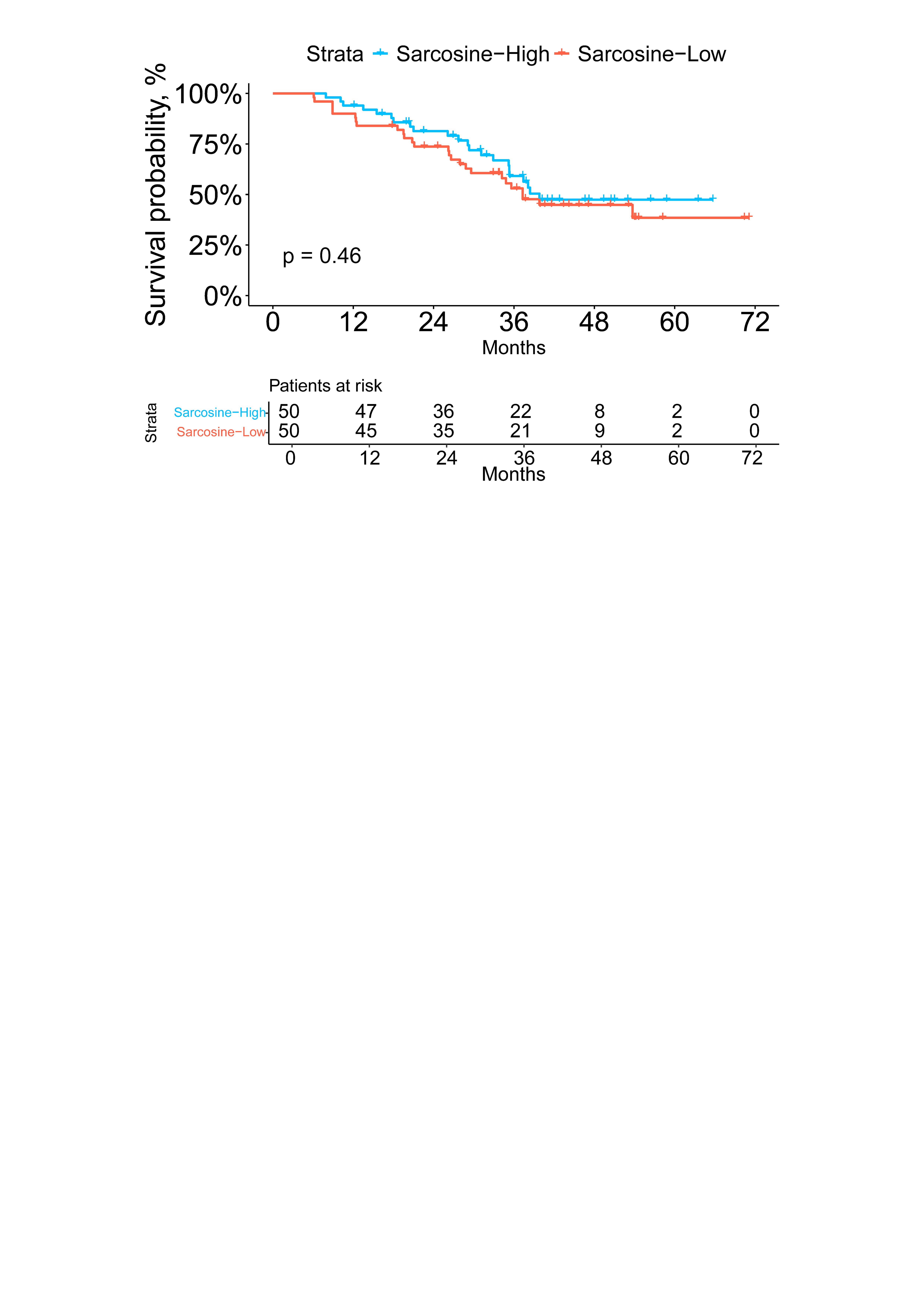
Fig S7**

**Figure S7 Clinical correlation of serum sarcosine with LUAD prognosis.**
Kaplan-Meier survival curves of 100 LUAD patients stratified by serum sarcosine levels.
