# Supplementary material for: Sarcosine sensitizes lung adenocarcinoma to chemotherapy by dual activation of ferroptosis via PDK4/PDHA1 signaling and NMDAR-mediated iron export
Source: Exp Hematol Oncol. 2025 Apr 24;14:60. doi: 10.1186/s40164-025-00657-0 (PMC12023509; doi:10.1186/s40164-025-00657-0)
Supplement: Supplementary file 3 — Supplementary Material 3 [file 40164_2025_657_MOESM3_ESM.docx]

**Supplementary Table 1**

**Sequences of sgRNA**

| **Target** | **sgRNA Sequence** | **Source** |
| --- | --- | --- |
| sgRNA-PDK4 | 5’- TCAGCATCCGAGTAGAAATA -3’ | Genechem |
| sgRNA-SLC40A1 | 5’- CAGAACGTACTCCACGCACA -3’ | Genechem |
| sgRNA-Ctrl | 5’-CGCTTCCGCGGCCCGTTCAA-3’ | Genechem |

**Protein sequences of PDK4-ΔAdom3**

| **Target** | **Protein Sequence** | **Source** |
| --- | --- | --- |
| PDK4-ΔAdom3 | MKAARFVLRSAGSLNGAGLVPREVEHFSRYSPSPLSMKQLLDFGSENACERTSFAFLRQELPVRLANILKEIDILPTQLVNTSSVQLVKSWYIQSLMDLVEFHEKSPDDQKALSDFVTLIKVRNRHHNVVPTMAQGIIEYKDACTVDPVTNQNLQYFLDRFYMNRISTRMLMNQHILIFSQTGNPSHIGSIDPNCDVVAVVQDAFECSRMLCDQYYLSSPELKLTQVNGKFPDQPIHIVYVPSHLHHMLFELFKNAMRATVEHQENQPSLTPIEVIVVLGKEDLTIKISDRGGGVPLRIIDRLFSYTYSTAPTPVMDNSRNAPLAGFGYGLPISRLYAKYFQGDLNLYSLSGYGTDAIIYLKALSSESIEKLPVFNKSAFKHYQMSSEADDWCIPSREPKNLAKEVAM | Genechem |

| **Sequences of shRNA** | **Target sequence** | **Source** |
| --- | --- | --- |
| MXD3-sh1 | 5’- GGUCAGUGCACAAUGAACUTT-3’ | Genechem |
| MXD3-sh2 | 5’- AGAGGCCGAGCAUGGUUAUTT-3’ | Genechem |

| **Primers for**  **qRT-PCR** | **Sequence** | **Source** |
| --- | --- | --- |
| ActinB | F: 5’-CTGGGACGACATGGAGAAAA -3’  R: 5’-AAGGAAGGCTGGAAGAGTGC -3’ | Sangon Biotech |
| MXD3 | F: 5’-CGCTCAGACTCAGACCAAGAGGA-3’  R: 5’-TCCATTCCAACAGGTGACTCCGA -3’ | Sangon Biotech |
| PDK4 | F: 5’- TGTCATTGGCAAGAGGAAGAACTG -3’  R: 5’- CCATTACCAGAAGCACCACAACAC -3’ | Sangon Biotech |
| SLC40A1 | F: 5’- TCTCCTACTACAACCAGCCTGTGTT-3’  R: 5’- TGAGATCAGACCTGTCCGAACCAA-3’ | Sangon Biotech |
| HLX | F: 5’- GTTCTGAAGGCGAGGCTGAGAG -3’  R: 5’- CACTGGTGCTGCTGCTACTAAGA -3’ | Sangon Biotech |
| HSF4 | F: 5’- AGTCTGCTGCCTCCGATGCT -3’  R: 5’- GCTCCATGTCCAAGTCCATCAGG -3’ | Sangon Biotech |
| SCX | F: 5’- GAGAACACCCAGCCCAAACAGAT -3’  R: 5’- GCCACCTCCTAACTGCGAATCG -3’ | Sangon Biotech |

| **Primary antibodies**  **for western blot** | **Producer** | **Catalogue number** | **Dilution** |
| --- | --- | --- | --- |
| ActinB | Beyotime | AF5001 | 1:2000 |
| phospho-PDHA1 (Ser293) | Abways | CY7247 | 1:2000 |
| PDHA1 | Abways | CY6656 | 1:2000 |
| p-AMPKα (Thr172) | Cell Signaling Technology | 50081S | 1:2000 |
| AMPKα | Cell Signaling Technology | 5832S | 1:2000 |
| PDK4 | AiFang Biological | AFW3069 | 1:1000 |
| MXD3 | Abways | DF6701 | 1:2000 |
| GRIN1 | Cell Signaling Technology | 5704 | 1:1000 |
| SLC40A1 | A14884 | Abclonal | 1:1000 |

| **Primary antibodies**  **for IHC** | **Producer** | **Catalogue number** | **Dilution** |
| --- | --- | --- | --- |
| anti-4-HNE | Abcam | ab48506 | 1:50 |
| anti-MXD3 | Abways | DF6701 | 1:100 |
| anti-SLC40A1 | A14884 | Abclonal | 1:100 |

| **Primers for ChIP-qPCR** |  | **Source** |
| --- | --- | --- |
| SLC40A1 primer 1 | F: 5’- GCTGACTTAGCCACTCTTTCCACAA -3’  R: 5’- GACAGGTCCTCAACAAGGCACAA -3’ | Sangon Biotech |
| SLC40A1 primer 2 | F: 5’- AACCGCTTCCATAAGGCTTTGC -3’  R: 5’- AGACTACAACGACGACTTTGGC -3’ | Sangon Biotech |

| **Primary antibodies**  **for multi-plex IHC (mIHC)** | **Producer** | **Catalogue number** | **Dilution** |
| --- | --- | --- | --- |
| phospho-PDHA1 (Ser293) | Abways | CY7247 | 1:100 |
| PDHA1 | Abways | CY6656 | 1:100 |
| 4-HNE | Abcam | ab48506 | 1:50 |
| PDK4 | AiFang Biological | AFW3069 | 1:100 |
| MXD3 | Abways | DF6701 | 1:100 |

**Supplementary Table 2**

*Summary descriptives table by tumor sarcosine abundance of 120 LUAD patients*

|  | **High** | **Low** | **p-value** |
| --- | --- | --- | --- |
|  | ***N=60*** | ***N=60*** |  |
| **Age** |  |  | 0.576 |
| <60 | 26 (43.3%) | 22 (36.7%) |  |
| >60 | 34 (56.7%) | 38 (63.3%) |  |
| **Stage** |  |  | 0.102 |
| I+II | 48 (80.0%) | 39 (65.0%) |  |
| III+IV | 12 (20.0%) | 21 (35.0%) |  |
| **Gender** |  |  | 0.710 |
| Female | 37 (61.7%) | 34 (56.7%) |  |
| Male | 23 (38.3%) | 26 (43.3%) |  |
| **T** |  |  | 0.181 |
| 1 | 37 (61.7%) | 30 (50.0%) |  |
| 2 | 13 (21.7%) | 14 (23.3%) |  |
| 3 | 6 (10.0%) | 14 (23.3%) |  |
| 4 | 4 (6.67%) | 2 (3.33%) |  |
| **N** |  |  | 0.006 |
| 0 | 41 (68.3%) | 26 (43.3%) |  |
| 1 | 15 (25.0%) | 19 (31.7%) |  |
| 2 | 4 (6.67%) | 15 (25.0%) |  |

**Supplementary Table 3**

*Summary descriptives table by tumor sarcosine abundance of 41 LUAD patients received cisplatin-based chemotherapy*

|  | **High** | **Low** | **p-value** |
| --- | --- | --- | --- |
|  | ***N=21*** | ***N=20*** |  |
| **Age** |  |  | 1.000 |
| <60 | 7 (33.3%) | 6 (30.0%) |  |
| >60 | 14 (66.7%) | 14 (70.0%) |  |
| **Stage** |  |  | 0.239 |
| I+II | 10 (47.6%) | 5 (25.0%) |  |
| III+IV | 11 (52.4%) | 15 (75.0%) |  |
| **Gender** |  |  | 0.444 |
| Female | 14 (66.7%) | 10 (50.0%) |  |
| Male | 7 (33.3%) | 10 (50.0%) |  |
| **T** |  |  | 0.203 |
| 1 | 7 (33.3%) | 7 (35.0%) |  |
| 2 | 8 (38.1%) | 3 (15.0%) |  |
| 3 | 3 (14.3%) | 8 (40.0%) |  |
| 4 | 3 (14.3%) | 2 (10.0%) |  |
| **N** |  |  | 0.124 |
| 0 | 3 (14.3%) | 4 (20.0%) |  |
| 1 | 14 (66.7%) | 7 (35.0%) |  |
| 2 | 4 (19.0%) | 9 (45.0%) |  |

**Supplementary Table 4**

*Summary descriptives table by serum sarcosine abundance of 100 LUAD patients*

|  | **High** | **Low** | **p-value** |
| --- | --- | --- | --- |
|  | ***N=50*** | ***N=50*** |  |
| Age: |  |  | 0.412 |
| <60 | 22 (44.0%) | 17 (34.0%) |  |
| >60 | 28 (56.0%) | 33 (66.0%) |  |
| Stage: |  |  | 0.035 |
| I+II | 43 (86.0%) | 33 (66.0%) |  |
| III+IV | 7 (14.0%) | 17 (34.0%) |  |
| Gender: |  |  | 1.000 |
| Female | 28 (56.0%) | 28 (56.0%) |  |
| Male | 22 (44.0%) | 22 (44.0%) |  |
| T: |  |  | 0.182 |
| 1 | 32 (64.0%) | 26 (52.0%) |  |
| 2 | 8 (16.0%) | 11 (22.0%) |  |
| 3 | 10 (20.0%) | 9 (18.0%) |  |
| 4 | 0 (0.00%) | 4 (8.00%) |  |
| N: |  |  | 0.055 |
| 0 | 34 (68.0%) | 24 (48.0%) |  |
| 1 | 13 (26.0%) | 16 (32.0%) |  |
| 2 | 3 (6.00%) | 10 (20.0%) |  |
